# Structural and Electrochemical Properties of Type VIII Ba_8_Ga_16−δ_Sn_30+δ_ Clathrate (δ ≈ 1) during Lithiation

**DOI:** 10.1021/acsami.1c07240

**Published:** 2021-09-03

**Authors:** Andrew Dopilka, Amanda Childs, Alexander Ovchinnikov, Ran Zhao, Svilen Bobev, Xihong Peng, Candace K. Chan

**Affiliations:** † Materials Science and Engineering, School for Engineering of Matter, Transport and Energy, Arizona State University, P.O. Box 876106, Tempe, Arizona 85827, United States; ‡ Department of Chemistry and Biochemistry, University of Delaware, Newark, Delaware 19716, United States; § Department of Materials and Environmental Chemistry, Stockholm University, Svante Arrhenius väg 16 C, 10691 Stockholm, Sweden; ∥ School of Molecular Sciences, Arizona State University, P.O. Box 871604, Tempe, Arizona 85287, United States; ⊥ College of Integrative Sciences and Arts, Arizona State University Polytechnic Campus, Mesa, Arizona 85212, United States; # Department of Heterogenous Catalysis, Max-Planck-Institut für Kohlenforschung, Kaiser-Wilhelm-Platz 1, 45470 Mülheim an der Ruhr, Germany

**Keywords:** clathrate, amorphous, pair distribution function, density functional theory, lithium, energy storage

## Abstract

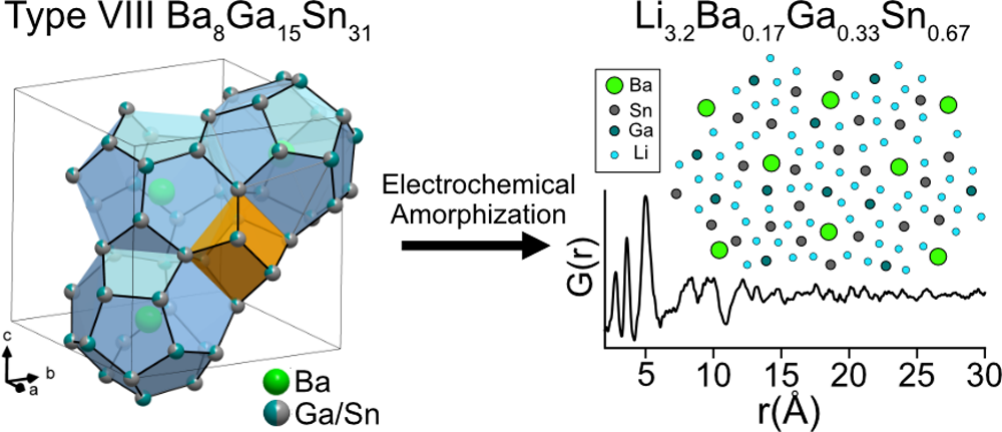

Clathrates of the tetrel (Tt = Si, Ge, Sn) elements are host–guest structures that can undergo Li alloying reactions with high capacities. However, little is known about how the cage structure affects the phase transformations that take place during lithiation. To further this understanding, the structural changes of the type VIII clathrate Ba_8_Ga_16−δ_Sn_30+δ_ (δ ≈ 1) during lithiation are investigated and compared to those in β-Sn with *ex situ* X-ray total scattering measurements and pair distribution function (PDF) analysis. The results show that the type VIII clathrate undergoes an alloying reaction to form Li-rich amorphous phases (Li_*x*_Ba_0.17_Ga_0.33_Sn_0.67_, *x* = 2–3) with local structures similar to those in the crystalline binary Li–Sn phases that form during the lithiation of β-Sn. As a result of the amorphous phase transition, the type VIII clathrate reacts at a lower voltage (0.25 V *vs* Li/Li^+^) compared to β-Sn (0.45 V) and goes through a solid-solution reaction after the initial conversion of the crystalline clathrate phase. Cycling experiments suggest that the amorphous phase persists after the first lithiation and results in considerably better cycling than in β-Sn. Density functional theory (DFT) calculations suggest that topotactic Li insertion into the clathrate lattice is not favorable due to the high energy of the Li sites, which is consistent with the experimentally observed amorphous phase transformation. The local structure in the clathrate featuring Ba atoms surrounded by a cage of Ga and Sn atoms is hypothesized to kinetically circumvent the formation of Li–Sn or Li–Ga crystalline phases, which results in better cycling and a lower reaction voltage. Based on the improved electrochemical performance, clathrates could act as tunable precursors to form amorphous Li alloying phases with novel electrochemical properties.

## 1 Introduction

Tetrel (Tt = Si, Ge, Sn) clathrates are host–guest materials with the potential for unique properties. While previously associated predominately with their thermoelectric,^1,2^ superconducting,^3,4^ and optical properties,^5−7^ the rich structural and compositional space of clathrates^1^ provides wide opportunities for tuning desirable characteristics for their application as next-generation anodes in Li-ion batteries. In tetrel clathrates, a cage framework of covalently bonded Tt elements encapsulates alkali and alkaline earth metal guest atoms. Clathrates can form in various crystal structures with defects such as guest atom vacancies/substitutions and framework vacancies/substitutions,^8^ leading to a wide design space. In recent years, our group and others have investigated the electrochemical properties of several clathrates comprising silicon^9−16^ and germanium^17−19^ frameworks.

We recently used X-ray pair distribution function (PDF) analysis to evaluate the local and long-range structure of the intermediates that form during room-temperature lithiation of Ba_8_Ge_43_ and Ba_8_Al_16_Ge_30_ clathrates, which both adopt the type I structure but contain vacancies or Al atoms, respectively, on the Ge framework.^19^ The PDF analysis showed that the lithiation of the clathrates proceeded through amorphous phase transformations, different from those in diamond cubic Ge (α-Ge), which often progress through crystalline phases.^20^ We hypothesized that during the lithiation process, the Ba atoms act as “pillars” that kinetically prevent long-range ordering of the regions rich in Li–Ge bonding, which results in suppression of crystalline phase formation. In these regions, the amorphous Li–Ge phases showed similar local structuring to Li–Ge crystalline phases of comparable composition, which was supported by the low-temperature amorphous-to-crystalline transformation observed with *in situ* PDF heating studies. Despite the destruction of the crystalline clathrate structure after lithiation, the PDF analysis showed that the cage-like local structure is preserved after delithiation. A consequence of this amorphous phase transformation is that lithium reacts *via* a solid-solution mechanism and at a lower voltage *vs* Li/Li^+^ relative to α-Ge, which are both beneficial properties for Li-ion battery anodes.

**Figure 1:**
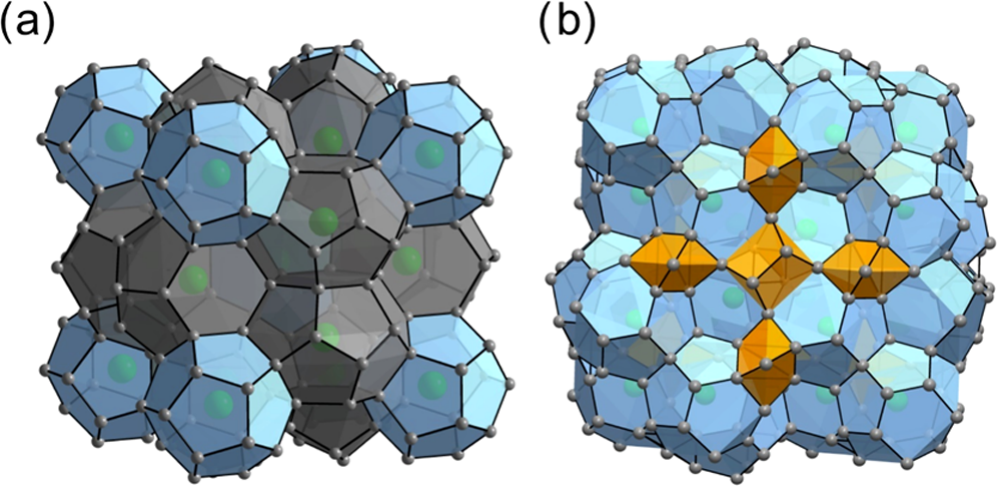
Crystal structures of (a) type I and (b) type VIII clathrates with ideal formulas M_8_Tt_46_. The gray atoms represent the Tt framework atoms and the green atoms represent the M guest atoms. The dodecahedra (Tt_20_) and distorted dodecahedra are shaded in blue, the tetrakaidecahedra (Tt_24_) in the type I structure are shaded in gray, and the voids in the type VIII structure are shaded in orange.

To further understand how the clathrate structure and composition affect its electrochemical reactivity with Li, herein we investigate the lithiation of the type VIII clathrate, which has a different but closely related structure than the previously investigated Ba–Ge clathrates with type I structure (Figure 1a). Both clathrate structures can be described with the same ideal formula, M_8_Tt_46_ (M = guest atom such as Na, Ba, *etc.*), but display different crystal chemistry.^21^ The type VIII clathrate crystal structure is described in an *I*4̅3*m* space group and is composed of face-sharing distorted dodecahedra (Tt_20_) filled by M-atoms and smaller voids (Figure 1b).^22,23^ This is notably different from the type I structure, which is composed of six larger tetrakaidecahedra (Tt_24_) and two pentagonal dodecahedra (Tt_20_) per formula unit.^2^ The polyhedra of the type VIII clathrate can be described as a strongly distorted version of the perfect dodecahedra found in the type I structure.^21^ Different from the Tt_20_ dodecahedra in type I clathrates, which contain only pentagonal faces, the Tt_20_ distorted dodecahedra in type VIII clathrates contain three, four, and five atom faces, which form six-membered and five-membered rings. In our previous studies, we identified Li migration through hexagonal faces as being associated with much lower-energy barriers compared to migration through pentagonal faces.^24^ Therefore, investigating the possibility of topotactic Li insertion through the six-membered rings of the distorted dodecahedra in the type VIII clathrate structure is of interest. In addition, the voids in the type VIII structure could provide a unique access point for Li into the polyhedral network, which has previously not been investigated.

Herein, we investigate the properties of type VIII clathrate Ba_8_Ga_16−δ_Sn_30+δ_ (δ ≈ 1), where a partial substitution of Sn for Ga atoms occurs on all framework sites. In an extension of our previous analysis comparing the lithiation properties in type I clathrate Ba_8_Ge_43_□_3_ (□ = vacancy) and Ba_8_Al_16_Ge_30_ with those of α-Ge, herein, we report a comparative study of the lithiation properties of type VIII clathrate with β-Sn. We find that the type VIII clathrate decomposes during the reaction with Li to form a highly lithiated amorphous phase and displays electrochemical properties distinct from those observed during lithiation of the Sn and Ga elemental phases. This is attributed to the assembly of Sn and Ga into the clathrate structure with Ba guest atoms, suggesting the possibility for novel amorphous alloy phases originating from clathrate precursors for anode materials.

## 2 Experimental Methods

The clathrate was prepared in a Sn flux using conditions that favor the type VIII over type I clathrate structure as described previously^23^ and the composition was confirmed by single-crystal X-ray diffraction (XRD). More details can be found in the Supporting Information. Slurries containing the active material (type VIII clathrate or commercially obtained β-Sn powder), carbon black, and binder were prepared and coated onto the copper foil (see the Supporting Information). These composite electrodes were then lithiated galvanostatically in half-cells with lithium metal and then extracted for *ex situ* measurements. Synchrotron X-ray pair distribution (PDF) measurements were conducted at Diamond Light Source (Didcot, U.K.) at the I15-I dedicated PDF beamline with λ = 0.161669 Å. The atomic PDF, *G*(*r*), as defined by Billinge et al.,^25^ was generated from the total scattering data using PDFgetx3 within the xPDF suite software package.^26,27^ The first-principles density functional theory (DFT) calculations were performed in a similar manner to our previous work^13,16,18,24^ and are described in more detail in the Supporting Information. The Gibbs free-energy change, average lithiation voltage, and formation energy were calculated as described previously.^13,18,24^ The climbing image nudged elastic band (NEB) method was used to calculate the Li migration barriers.^28^ More detailed descriptions of the synthesis, electrochemical, synchrotron measurements, PDF analysis, and DFT calculations are in the Supporting Information.

## 3 Results

### 3.1 Type VIII Ba–Ga–Sn Clathrate Structure

**Figure 2:**
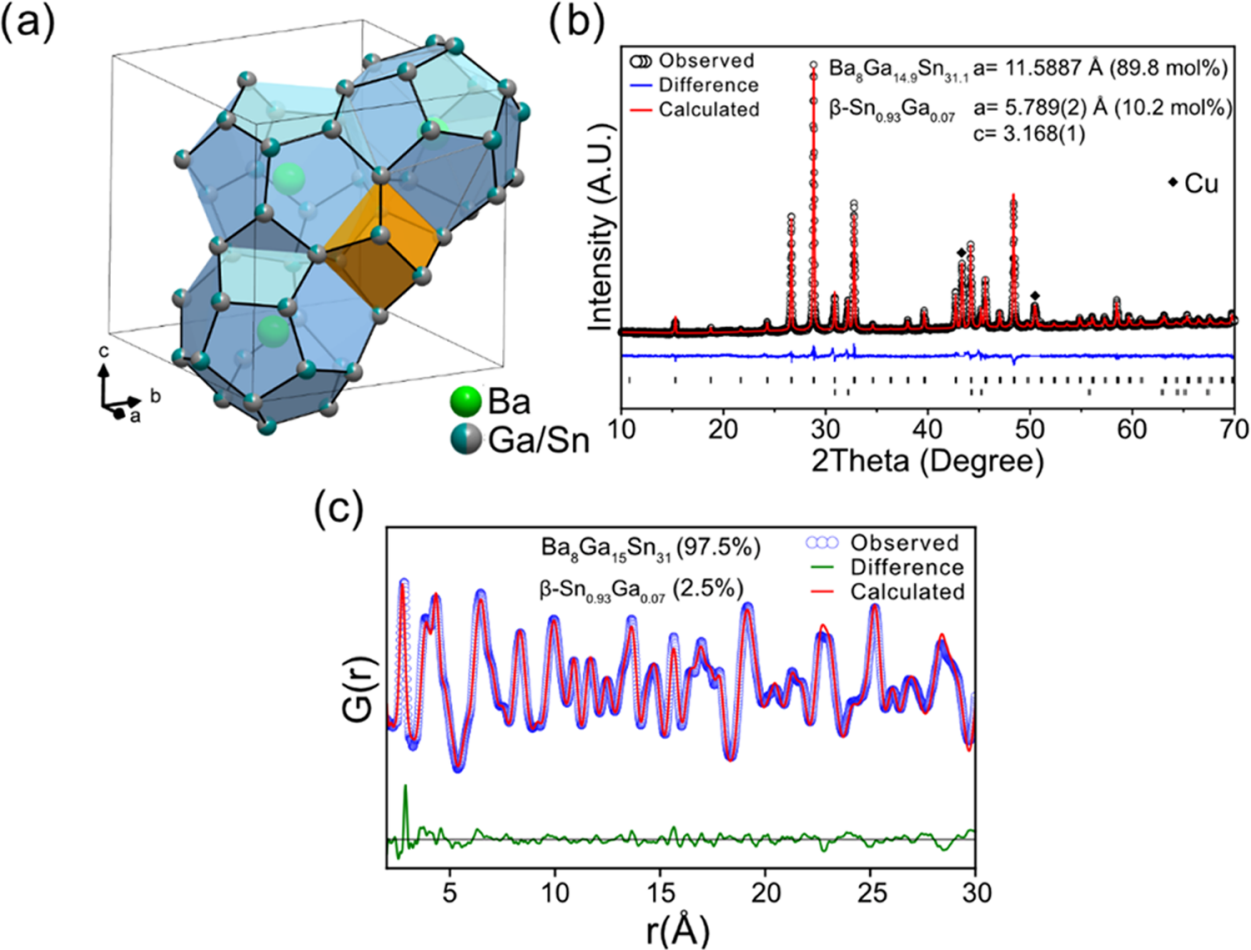
(a) Crystal structure of type VIII Ba_8_Ga_15_Sn_31_ clathrate derived from the single-crystal refinement (Tables S1 and S2). The relative fractions of gray/blue spheres represent the occupancy of Sn/Ga atoms on framework sites, and the green spheres represent the Ba atoms. The distorted dodecahedra are shaded in blue, while the void is shaded in orange. (b) Rietveld refinement of the PXRD pattern of the electrode used in the electrochemical experiments. The lattice and atomic parameters of Ba_8_Ga_14.9_Sn_31.1_ were fixed to that found in the single-crystal refinement. The top set of tick marks corresponds to the Ba_8_Ga_14.9_Sn_31.1_ phase (*I*4̅3*m*), and the bottom set corresponds to β-Sn_0.93_Ga_0.07_ (*I*4_1_/*amd*). Refinement residuals: χ^2^ = 1.58, *R*_p_ = 0.042, and w*R*_p_ = 0.057. (c) PDF refinement (*R*_w_ = 15.1%) of pristine Ba_8_Ga_15_Sn_31_ using the structural model derived from the single-crystal refinement (Tables S1 and S2). Phase fractions are in terms of mol %.

Single-crystal XRD analysis of the products of the flux reaction (see Tables S1 and S2 for refinement and structural details) confirmed that the synthesized materials adopted the type VIII clathrate crystal structure with a refined composition of Ba_8_Ga_14.9_Sn_31.1(4)_; for simplicity, Ba_8_Ga_15_Sn_31_ is used as the composition for the subsequent electrochemical characterization. A close-up view of the crystal structure of Ba_8_Ga_15_Sn_31_ is shown in Figure 2a. Energy-dispersive X-ray spectroscopy (EDS) characterization of the single-crystal particles supports the composition derived from the single-crystal XRD refinement (Figure S1a). Laboratory powder XRD (PXRD) performed on the as-synthesized clathrate powders confirmed that the product mainly consisted of the type VIII clathrate along with other impurities (Figure S1b). To quantify the phase fractions, Rietveld refinement analysis was performed on the PXRD pattern of the electrode (Figure 2b). The diffraction pattern showed reflections corresponding to the type VIII clathrate phase along with a 10.2 mol % phase fraction of β-Sn_0.93_Ga_0.07_, which likely originates from the residual Sn flux from the synthesis. The presence of Ga substitution in the β-Sn phase was determined based on the lattice parameter, which matched well with a previous report (*a* = 5.784 Å, *c* = 3.168 Å for β-Sn_0.93_Ga_0.07_).^29^ Synchrotron PDF analysis was also used to quantify the amount of β-phase impurity in the clathrate sample. The PDF refinement of the pristine clathrate (Figure 2c and Table S3) showed that the PDF fit the structural model derived from the single-crystal structural refinements presented herein (Tables S1 and S2) with a fraction of β-Sn_0.93_Ga_0.07_ (2.5 mol %). The Rietveld and PDF refinements give different values for the phase fraction of β-Sn_0.93_Ga_0.07_ in the sample, which could originate from particle size effects from loading the powder into the capillary for PDF measurements. The PXRD pattern of the electrode is expected to be more representative of the phases present during electrochemical lithiation as the powder was well mixed in a slurry before casting the electrode.

### 3.2 Electrochemical Lithiation

To investigate the structures that form upon lithiation, the type VIII clathrate and β-Sn electrodes were electrochemically lithiated with similar amounts of Li and then subjected to total scattering experiments to obtain structure function and PDF plots. Scanning electron microscopy (SEM) imaging of the electrodes prior to lithiation (Figure S2) showed that the type VIII clathrate was composed of irregularly shaped particles 2–20 μm in size with small spherical β-Sn_0.93_Ga_0.07_ impurities, while the β-Sn electrode prepared for comparison was composed of 1–5 μm spherical particles.

**Figure 3:**
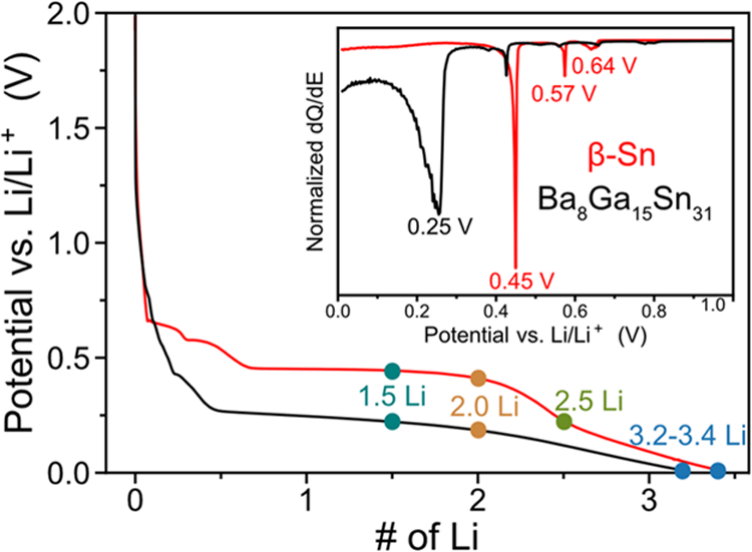
Voltage profile and corresponding d*Q*/d*E* plot of the lithiation of Ba_8_Ga_15_Sn_31_ (black) and β-Sn (red) at 12.5 and 25 mA/g, respectively, with a voltage cutoff of 10 mV *vs* Li/Li^+^. The circles represent the points where cells were disassembled for *ex situ* total scattering measurements. The # of Li is normalized to the amount of nonalkali/alkaline earth metal atoms.

The voltage profiles and corresponding d*Q*/d*E* plots for the first lithiation of β-Sn and Ba_8_Ga_15_Sn_31_ are presented in Figure 3, with teal, orange, green, and blue points representing the compositions at which samples were collected for total scattering measurements. The voltages and capacities of the cells at these points in the lithiation process are presented in Table S4. Note that the number of Li added to each electrode is normalized to the amount of Sn or (Ga + Sn) atoms in each starting compound.

The room-temperature voltage profile of β-Sn is characterized by three voltage plateaus and then a sloped curve until reaching a cutoff voltage of 10 mV *vs* Li/Li^+^. The plateaus at 0.64 and 0.57 V *vs* Li/Li^+^ (represented as peaks in the d*Q*/d*E* plot) have been assigned to the formation of Li_2_Sn_5_^30^ and LiSn,^31^ respectively.^32−37^ The plateau at 0.45 V has been previously assigned to an amorphous transition on the basis of *in situ* XRD analysis,^33,35^ while an *in operando* NMR study of the lithiation of Sn nanoparticles recently assigned this plateau to the conversion of LiSn to Li_7_Sn_3_ and then Li_13_Sn_5_.^36^ After the plateau at 0.45 V, the voltage profile is more sloped and reaches a final composition of 3.4 Li per Sn, which contains less Li than the theoretical maximum based on the most Li-rich compound (previously Li_22_Sn_5_ but later identified as Li_17_Sn_4_^38^) from the Li–Sn phase diagram but is similar to previous experimental reports.^32,37^ It has been proposed that disordered Li_17_Sn_4_ forms in this sloped voltage region^39,40^ by X-ray diffraction analysis, as well as Li_7_Sn_2_*via* NMR measurements.^36^

The lithiation of the type VIII clathrate is characterized by a voltage plateau starting at 0.25 V (seen as a peak in the d*Q*/d*E* plot) and then a sloping profile. We assign the process occurring at 0.25 V to the lithiation of the type VIII clathrate phase. The sample reacts with around 3.2 Li per (Ga + Sn) atom, which suggests that the clathrate is being converted into a phase containing high Li content. We note that this composition is an overestimate due to the presence of β-Sn_0.93_Ga_0.07_ as an impurity (which is identified by the small peaks from 0.40 to 0.64 V in the d*Q*/d*E* plot that match those in the β-Sn electrode). Additionally, there is likely the formation of a solid electrolyte interphase (SEI) due to electrolyte reduction in the first lithiation; both of these processes are expected to consume lithium.

### 3.3 Structure Function Patterns

**Figure 4:**
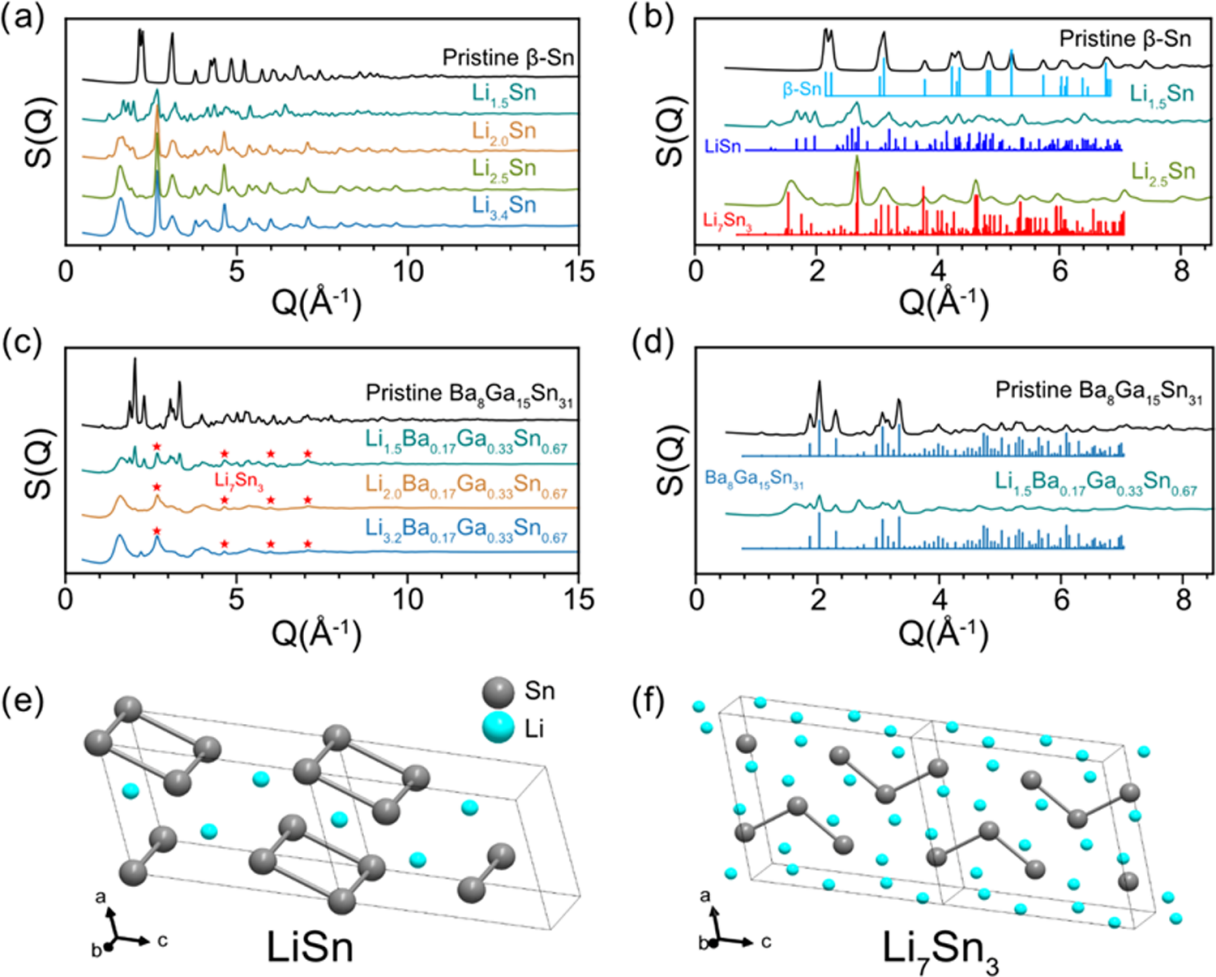
(a) Structure function plots for pristine β-Sn and lithiated β-Sn electrodes. (b) Comparison of structure function plots for β-Sn, Li_1.5_Sn, and Li_2.5_Sn with calculated reference patterns for β-Sn, LiSn, and Li_7_Sn_3_. (c) Structure function plots for pristine Ba_8_Ga_15_Sn_31_ and lithiated clathrate electrodes. (d) Comparison of structure function plots for pristine clathrate and Li_1.5_Ba_0.17_Ga_0.33_Sn_0.67_ with reference pattern for Ba_8_Ga_15_Sn_31_ (from single-crystal refinement; see Tables S1 and S2). Crystal structures of intermediate phases identified in the lithiation of β-Sn: (e) LiSn (data taken from ref (31)) and (f) Li_7_Sn_3_ (data taken from ref (41)).

Structure function patterns, *S*(*Q*), derived from the total scattering patterns for the β-Sn and Ba_8_Ga_15_Sn_31_ electrodes before and after lithiation are presented in Figure 4a,c, respectively. The structure function plots allow for the observation of Bragg peaks at low scattering angles and to tentatively identify the major crystalline phases present in the sample by comparison with calculated reference patterns. To aid comparison, the plots for the lithiated electrodes are labeled at the compositions indicated by the points in Figure 3, *i.e.*, Li_*x*_Sn (*x* = 1.5, 2.0, 2.5, 3.4) for the lithiated β-Sn compositions and Li_*x*_Ba_0.17_Ga_0.33_Sn_0.67_ (*x* = 1.5, 2.0, 3.2) for the lithiated clathrate compositions (Li amounts are normalized to the amount of nonalkali/alkaline earth metal atoms). Figure 4b,d shows the structure function patterns with the calculated reference patterns of the identified phases. For the β-Sn electrodes (Figure 4a), reflections corresponding to β-Sn disappear and a series of other reflections appear as the Li composition increases during lithiation. At a composition of Li_1.5_Sn, reflections corresponding to LiSn (*P*2/*m*)^31^ are present, as seen by the comparison of the calculated reference pattern with the structure function (Figure 4b). In the Li_2.0_Sn pattern, the reflections corresponding to LiSn (*P*2/*m*)^31^ decrease in favor of another set of reflections that are assigned to Li_7_Sn_3_ (*P*2_1_/*m*),^41^ which suggests that a two-phase reaction is occurring in this part of the lithiation process. Crystal structures of LiSn and Li_7_Sn_3_ are shown in Figure 4e,f and demonstrate how the Sn square units in LiSn are broken up to form Sn trimers in the Li_7_Sn_3_ phase. At a composition of Li_2.5_Sn, the sample appears to be mainly composed of Li_7_Sn_3_ based on the comparison to the calculated pattern (Figure 4b). For the electrode with composition Li_3.4_Sn, the pattern is similar to that for Li_2.5_Sn, suggesting that Li_7_Sn_3_ is still present but with slight changes to some of the intensities of the reflections. Overall, the structure function patterns confirm that the lithiation of β-Sn proceeds through crystalline phase transformations.

For the structure function patterns of the type VIII clathrate (Figure 4c), the pristine (unlithiated) sample shows reflections matching the calculated pattern for the type VIII Ba_8_Ga_15_Sn_31_ structure obtained from the single-crystal refinement (Figure 4d), consistent with the lab PXRD results. Lithiation to a composition of Li_1.5_Ba_0.17_Ga_0.33_Sn_0.67_ resulted in a decrease in the intensity of reflections corresponding to Ba_8_Ga_15_Sn_31_ and the emergence of several broad peaks. No other significant reflections are present, suggesting that the broad peaks originate from an amorphous phase. At a composition of Li_2.0_Ba_0.17_Ga_0.33_Sn_0.67_, the pattern only displays the broad reflections associated with the amorphous phase and there are no significant reflections past 5 Å^–1^, suggesting that the type VIII clathrate has been fully converted into the amorphous phase. Low-intensity reflections (Figure 4c, marked as red stars) are observed that match those in the calculated pattern for Li_7_Sn_3_; the presence of these reflections is attributed to the lithiation of the β-Sn_0.93_Ga_0.07_ impurity phase to form Li_7_Sn_3_. For Li_3.2_Ba_0.17_Ga_0.33_Sn_0.67_, the structure function pattern is similar to the pattern for Li_2.0_Ba_0.17_Ga_0.33_Sn_0.67_ but with slightly shifted intensities for the broad peaks. Analysis of the structure function patterns of the intermediates formed during reaction of Li with Ba_8_Ga_15_Sn_31_ suggests that lithiation proceeds through the conversion of the clathrate phase to an amorphous phase.

### 3.4 Pair Distribution Function Analysis

By taking a Fourier transform of the reduced structure functions, the total scattering data can be analyzed in real space and allows for the observation of local structural changes through the pair distribution function (PDF).^25^ The PDF plot provides a weighted histogram of all atom–atom distances within a material and has been used to great effect to understand electrochemical alloying reactions.^19,20,42−45^ The calculated PDF patterns for Li–Sn crystalline phases and Ba_8_Ga_15_Sn_31_ discussed herein are presented in Figures S3 and S4, respectively.

**Figure 5:**
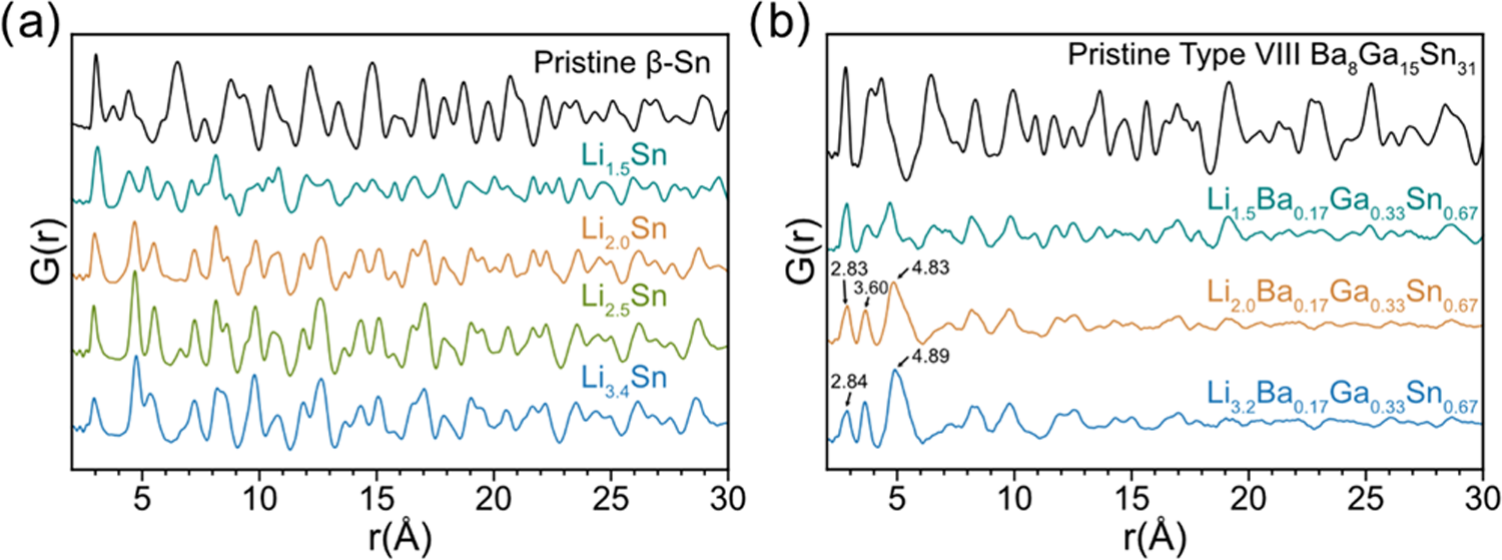
(a) *Ex situ* X-ray PDFs for pristine β-Sn, Li_1.5_Sn, Li_2.0_Sn, Li_2.5_Sn, and Li_3.4_Sn. (b) *Ex situ* X-ray PDFs for pristine Ba_8_Ga_15_Sn_31_, Li_1.5_Ba_0.17_Ga_0.33_Sn_0.67_, Li_2.0_Ba_0.17_Ga_0.33_Sn_0.67_, and Li_3.2_Ba_0.17_Ga_0.33_Sn_0.67_.

*Ex situ* PDF plots for the lithiated β-Sn and the type VIII clathrate electrodes are presented in Figure 5. The PDF refinement for the pristine β-Sn sample fit the structural model well, suggesting phase-pure starting material (Figure S5). The PDFs of the β-Sn after lithiation were refined (Figure S6 and parameters in Table S5) from 2 to 15 Å to LiSn, Li_7_Sn_3_, and Li_7_Sn_2_ based on the expected reaction products from the structure function plots (Figure 4a,b) and from previously reported assignments.^32^ The PDF for the electrode lithiated to a composition of Li_1.5_Sn was fit to LiSn and Li_7_Sn_3_, the expected products at this composition, and resulted in a relatively good fit (*R*_w_ = 15.9%) with phase fractions of 70 mol % LiSn and 30 mol % Li_7_Sn_3_ (Figure S6a). For the Li_2.0_Sn sample, the PDF was fit to 82 mol % Li_7_Sn_3_ and 18 mol % LiSn with an *R*_w_ of 11.5% (Figure S6b). The Li_2.5_Sn pattern was fit well by considering Li_7_Sn_3_ as the sole phase (*R*_w_ = 13.9%; Figure S6c), while the Li_3.4_Sn PDF was fit well to a mixture of 53 mol % Li_7_Sn_3_ and 47 mol % Li_7_Sn_2_ (*R*_w_ = 19.9%; Figure S6d). Interestingly, when the fit range was increased to 30 Å, the fit was worse for PDFs containing Li_7_Sn_3_ (see Figure S6e), with the high-*r* correlations from 20 to 30 Å not captured well by the Li_7_Sn_3_ model. Another study showed that Li_7_Sn_3_ synthesized *via* ball-milling and annealing showed a good fit of the PDF to the Li_7_Sn_3_ structure from 2 to 30 Å,^43^ suggesting that the deviation from the Li_7_Sn_3_ structural model observed in our results (Figure S6e) might be related to the electrochemical formation of Li_7_Sn_3_ at room temperature. Overall, the PDF analysis shows that the lithiation of β-Sn proceeds through crystalline phase transformations in which the Sn clusters in LiSn (Figure 4e) are transformed into smaller units until Sn dumbbells/trimers and single Sn atoms surrounded by Li atoms (as seen in Li_7_Sn_3_ and Li_7_Sn_2_; see Figures 4f and S6f, respectively) are the dominant features present at the end of lithiation.

In the case of the clathrate samples, since the intensity of X-ray PDF correlations is dependent on the atomic number of the elements, the Sn atomic correlations are expected to contribute the most to the observed PDF patterns. The higher contribution of the Sn–Sn and Sn–Ga correlations to the total PDF pattern for Ba_8_Ga_15_Sn_31_ can be readily seen in the calculated pattern in Figure S4. Inspection of this PDF shows that the first, second, and third correlations in the total PDF pattern correspond to direct Sn/Ga–Sn/Ga bonding, Ba–Sn/Ga distances, and next-nearest neighbor Sn/Ga distances, respectively. The PDF results for the lithiation of the type VIII Ba_8_Ga_15_Sn_31_ (Figure 5b) show that lithiation of the clathrate results in decreased intensities and significant shifts in the correlations, which is indicative of phase transformations. Refinement of the PDF from Li_1.5_Ba_0.17_Ga_0.33_Sn_0.67_ showed that correlations corresponding to the pristine type VIII clathrate are still present, but the refinement could not capture all of the correlations at high-*r* values (10–30 Å) (Figure S7a). Adding Li_7_Sn_3_ to the refinement as a second phase results in a better fit with 30 mol % clathrate and 70 mol % Li_7_Sn_3_ (Figure S7b and Table S6). We attribute the presence of Li_7_Sn_3_ to originate from the lithiation of the β-Sn_0.93_Ga_0.07_ impurity in the clathrate sample. Li_7_Sn_3_ was chosen as the second phase for the refinement because it is the expected product from the lithiation of β-Sn at the voltage of this particular electrode (0.17 V); furthermore, the Bragg peaks in the structure function pattern of this sample showed reflections from both the clathrate and Li_7_Sn_3_ structures (Figure 4c). After fitting the PDF pattern to the clathrate and Li_7_Sn_3_ phases, three peaks appear in the difference plot at *r* values of 2.88, 3.60, and 4.70 Å (Figure S7b). We attribute the origins of these correlations to the lithiated amorphous phase that forms when lithium reacts with the clathrate. This result is similar to our previous PDF analysis of type I clathrate Ba_8_Ge_43_ after lithiation, where a similar treatment of the refinement revealed an amorphous phase with three peaks at low-*r* values in the difference plot.^19^ Based on the similar result and absence of other Bragg peaks appearing in the structure function pattern (Figure 4c), we attribute the peaks in the difference plot to an amorphous Li–Ba–Ga–Sn phase that coexists with the pristine (unlithiated) clathrate phase at this stage of the lithiation process.

Further lithiation of the type VIII clathrate to a composition of Li_2.0_Ba_0.17_Ga_0.33_Sn_0.67_ resulted in further decreases in the intensity of the high-*r* correlations (10 < *r* < 30 Å), suggesting the complete conversion of the crystalline Ba_8_Ga_15_Sn_31_ by this point, consistent with the structure function plots (Figure 4c). At low-*r* values (2 < *r* < 5 Å), three main correlations are present at 2.83, 3.60, and 4.83 Å. These correlations are at similar distances to those attributed to the amorphous phase in the PDF for Li_1.5_Ba_0.17_Ga_0.33_Sn_0.67_ (Figure S7b), suggesting that the amorphous phase continues to grow at the expense of the pristine clathrate phase. The low intensity of correlations past 15 Å indicates the absence of long-range order in the sample, consistent with the broad peaks in the structure function pattern (Figure 4c). Further lithiation to Li_3.2_Ba_0.17_Ga_0.33_Sn_0.67_ shows a similar PDF pattern as that for Li_2.0_Ba_0.17_Ga_0.33_Sn_0.67_, but the first correlation at 2.84 Å has a lower intensity relative to the correlation at 4.89 Å, an indication that Sn/Ga–Sn/Ga bonds are being broken to form more Sn/Ga single atoms. Furthermore, the presence of low-intensity correlations from 15 to 30 Å in both PDF patterns suggests the presence of a small amount of crystalline phase; we attribute this to possible Li–Sn crystalline phases formed from the reaction of the β-Sn_0.93_Ga_0.07_ impurity present in the clathrate starting material as described previously (Figure 2b).

**Figure 6:**
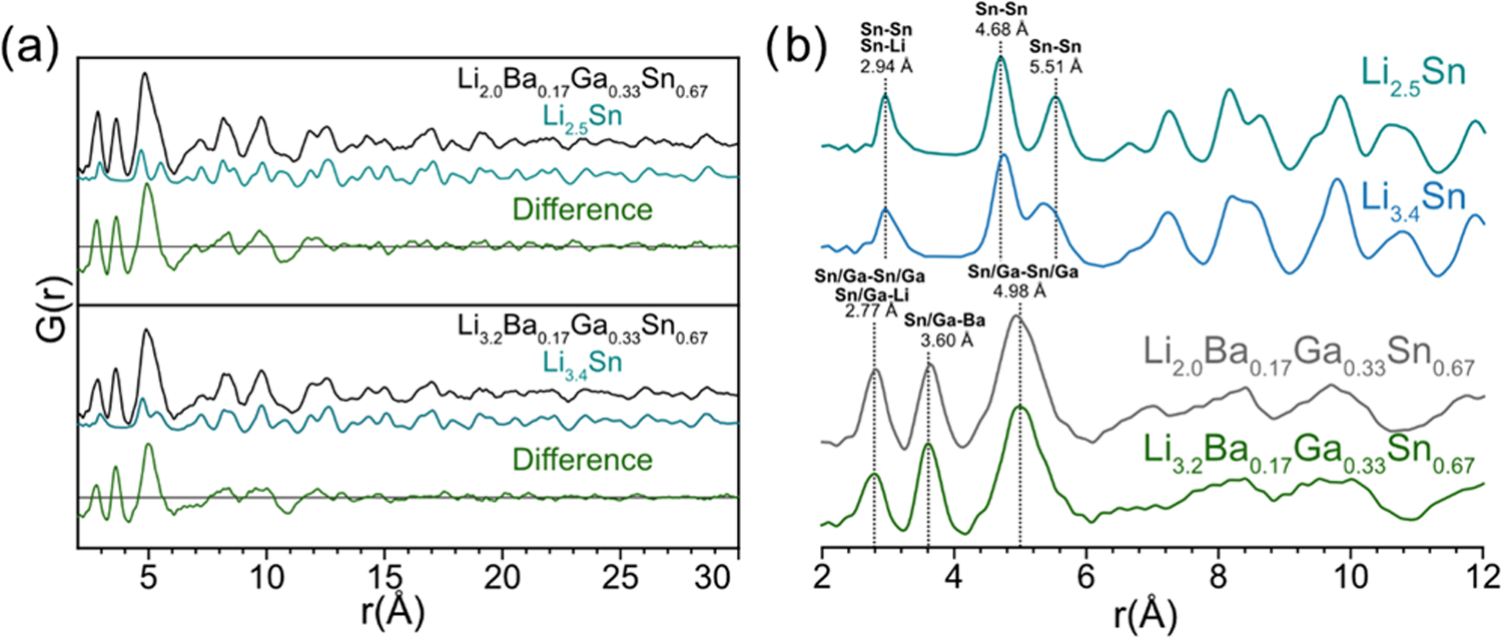
(a) Difference curves resulting from the subtraction of the Li–Sn PDF (crystalline impurity phase) from the PDF of the amorphous phase formed during lithiation of the clathrate. (b) Comparison of the lithiated PDFs for β-Sn and the type VIII clathrate with the indicated compositions. From the refinements, the electrode with composition Li_2.5_Sn was determined to contain Li_7_Sn_3_, while the electrode with composition Li_3.4_Sn contained a mixture of Li_7_Sn_3_ and Li_7_Sn_2_.

From these results, it appears that the Li–Sn crystalline phases that form upon lithiation of the β-Sn_0.93_Ga_0.07_ impurity contribute substantially to the PDF of the lithiated clathrate electrode, especially at high-*r* values. To better elucidate the local structure of the amorphous phases formed upon lithiation of the clathrate, the contribution of the residual crystalline Li–Sn compounds was removed by subtracting the appropriate experimental Li_*x*_Sn PDF from the PDFs of the lithiated clathrates. The results of this process are shown in Figure 6a. For the PDF of Li_2.0_Ba_0.17_Ga_0.33_Sn_0.67_, the subtraction was performed using the PDF taken from the Li_2.5_Sn electrode because both cells were lithiated to a similar voltage (Table S4). The experimental PDF was scaled to fit over the high-*r* correlations and then subtracted from the clathrate PDF. The correlations from the experimental Li_2.5_Sn PDF, which was refined to 100% Li_7_Sn_3_ (Figure S6c), matched the high-*r* correlations of the lithiated type VIII clathrate well, as seen by the low intensity in the difference curve from 15 < *r* < 30 Å (Figure 6a). Based on the good fit, we expect that the small amount of Ga alloying (7%) into the Sn phase (Figure 2b) did not significantly affect the structures of the lithiated phases compared to those of the pure Li–Sn compositions. A similar process was conducted for the PDF of Li_3.2_Ba_0.17_Ga_0.33_Sn_0.67_ but using subtraction of the PDF from Li_3.4_Sn. Attempts were made to refine the experimental patterns of Li_2.0_Ba_0.17_Ga_0.33_Sn_0.67_ and Li_3.2_Ba_0.17_Ga_0.33_Sn_0.67_ to the structure of Li_7_Sn_3_ (Figure S7c,d). The results showed a similar difference plot from 2 to 10 Å as those shown in Figure 6a, but the high-*r* (20–30 Å) correlations were not fit as well, consistent with the poor fit to Li_7_Sn_3_ observed in the refinement of the Li_2.5_Sn PDF from 20 to 30 Å (Figure S6e). The contributions from the impurity Li–Sn crystalline phases were dominant in the PDF above 6 Å, and by subtracting them from the clathrate PDFs, it gives a clearer picture of the features originating from the lithiated amorphous phase formed upon the reaction of lithium with the clathrate.

In Figure 6b, the PDF patterns for the β-Sn and type VIII clathrate samples after lithiation are compared. The lowest *r* values are at 2.94 and 2.77 Å, respectively. In many binary Li–Sn compounds, the first correlation in the PDF is a combination of direct Sn–Sn bonding and Li–Sn correlations (Figure S3). The Sn–Sn bond lengths in Li_7_Sn_3_ and Li_7_Sn_2_ are 3.00 and 2.95 Å, respectively, which matches the correlation at 2.94 Å and is slightly shorter than the Sn–Sn bond length in β-Sn of 3.11 Å. In comparison, the first correlation seen in the lithiated clathrate with a composition of Li_3.2_Ba_0.17_Ga_0.33_Sn_0.67_ (2.77 Å) is much shorter than the first correlation distances in β-Sn, Li_7_Sn_3_, and Li_7_Sn_2_ while slightly larger than the Sn–Ga bond length in the pristine type VIII clathrate structure (2.642–2.764 Å).^23^ This suggests that the Ga atoms are participating in bonding with Sn in the amorphous lithiated phase and thus results in a lower average bond length when compared to pure Sn–Sn bonding. The next correlation occurs at 3.60 Å for the clathrate-derived lithiated phases, which is similar to the Ba–Ga/Sn distances in the pristine clathrate phase (3.632–3.852 Å).^23^ The absence of this peak in the PDFs of the Li–Sn reference compounds (Figure S3), as well as the lithiated β-Sn samples, confirms that it is associated with Ba–Ga/Sn correlations. This Ba correlation is also notably similar to the Ba–Ge distance (3.44 Å) observed previously in the PDFs of amorphous Li–Ba–Ge phases formed after lithiation of type I Ba–Ge clathrates.^19^ The correlations at 4.68 and 5.51 Å in the Li_2.5_Sn PDF are associated with two different next-nearest neighbor Sn–Sn correlations in the Li_7_Sn_3_ phase (Figure S3b). In the clathrate sample, a single correlation is centered at 4.98 Å. The splitting of this peak into two correlations is a consequence of the parallel alignment of two different next-nearest-neighbor distances of Sn–Sn units (Sn trimers in the case of Li_7_Sn_3_; see Figures 4f and S8), while it becomes a single peak as the amount of isolated Sn atoms increases (as in Li_7_Sn_2_; see Figures S3c and S6f). The peak at 4.98 Å for the clathrate sample is expected to correspond to a similar type of the next-nearest-neighbor correlation; however, the peak is not split, which could be due to a disorder between Sn/Ga–Sn/Ga units in the amorphous phase or the presence of more isolated single atoms. We note that this single peak at 4.98 Å is a feature similar to that observed in the PDFs of amorphous lithiated Ba_8_Ge_43_^19^ and in lithiated nanoparticles of amorphous Ge and has been attributed to the absence of parallel alignment of adjacent dumbells.^43^ Another notable feature in the PDFs for both β-Sn and the clathrate materials is the decrease in intensity of the first correlation (2.7–2.9 Å) relative to the correlations from 4.7 to 5.2 Å as the Li content increases. This behavior is indicative of a breaking of direct Sn/Ga bonds resulting in more isolated Sn/Ga atoms surrounded by Li, which manifests as a single peak around 5 Å. This is seen in the PDF of Li_3.4_Sn (Figure 5a) where instead of completely two split peaks (as seen in Li_2.5_Sn at 4.68 and 5.51 Å), these correlations are starting to merge, which is an indication that the Sn trimers are being broken up in favor of single Sn atoms. Since a similar trend is observed for the clathrate sample, we suspect that Sn/Ga–Sn/Ga bonds are being broken in an analogous manner albeit *via* a more disordered arrangement.

Finally, after the subtraction of the Li–Sn contribution to the PDF pattern, broad peaks at around 6–12 Å are present in the PDFs of the lithiated clathrate electrodes at similar positions to the correlations in the PDFs of the Li–Sn compounds, suggesting the presence of analogous but disordered structures in the amorphous phases. We made a similar observation when comparing the PDFs of crystalline Li–Ge binary compounds with those of amorphous phases formed after lithiation of Ba_8_Ge_43_ in our previous work.^19^ There, we found from PDFs taken during *in situ* heating at 420–450 K that the amorphous lithiated Ba_8_Ge_43_ phases crystallized into binary Li–Ge phases. With *in situ* PDF, we demonstrated that the broad correlations from 6 to 10 Å in the amorphous sample were disordered analogues of similar correlations in the crystalline Li–Ge phases. We performed similar *in situ* PDF heating experiments from 300 to 420 K (Figure S9) of the amorphous Li_3.2_Ba_0.17_Ga_0.33_Sn_0.67_, but no significant crystallization events were observed over this temperature range, possibly due to the need for a higher temperature or the complication of Ga being present. Overall, the PDF results show that the local structure of the amorphous Li–Ba–Ga–Sn phase has similarities to those in the crystalline Li–Sn phases. The main difference in the structures is the lack of long-range order and the presence of the Ba–Ga/Sn correlation at 3.60 Å, suggesting that the Ba atoms are intermixed next to Sn/Ga atoms.

### 3.5 Galvanostatic Cycling

**Figure 7:**
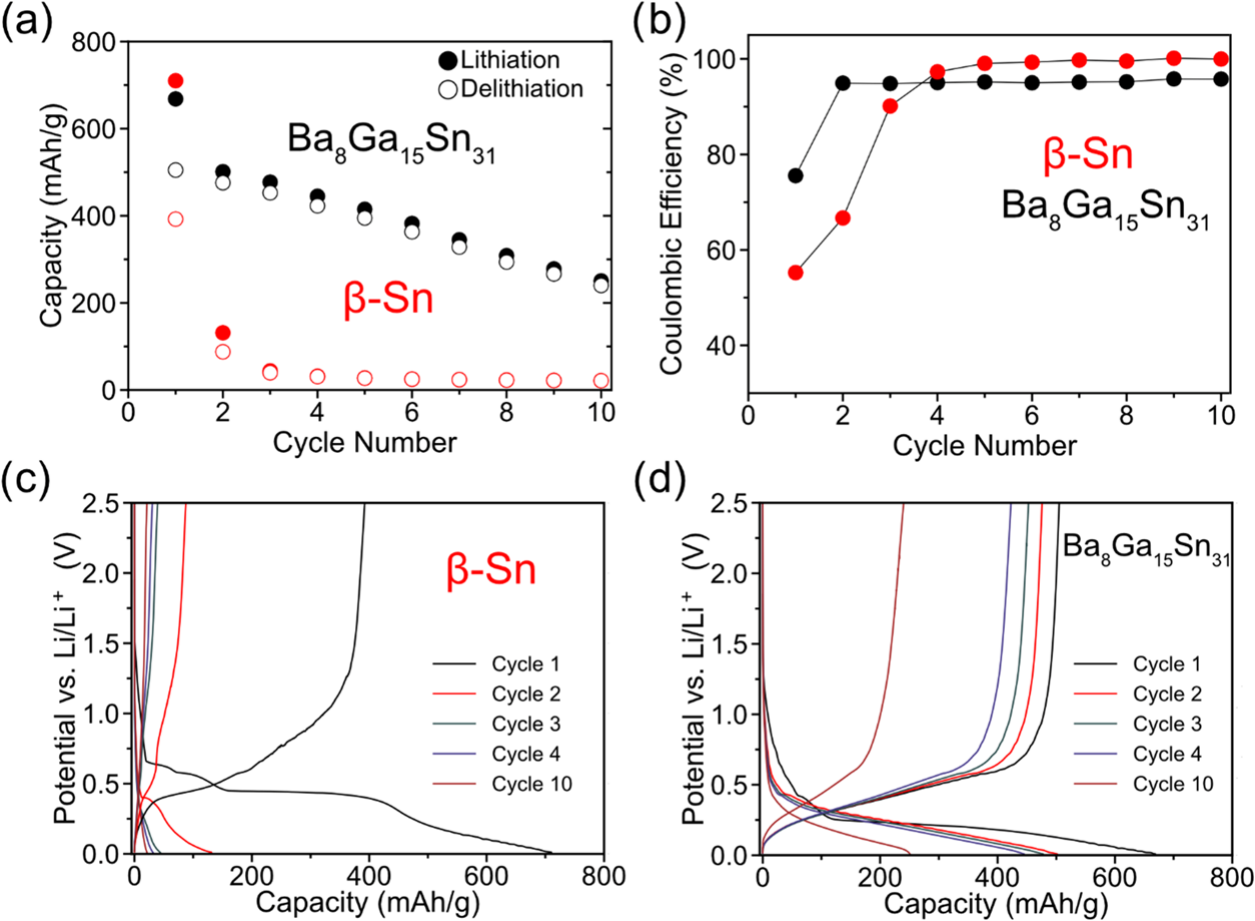
(a) Capacity and (b) Coulombic efficiency *vs* cycle number for β-Sn and type VIII Ba_8_Ga_15_Sn_31_ clathrate electrodes in half-cells cycled at 12.5 mA/g with a voltage range of 0.01–2.5 V *vs* Li/Li^+^. Voltage profiles for (c) β-Sn and (d) type VIII Ba_8_Ga_15_Sn_31_ clathrates.

Next, the effect of different lithiation mechanisms on cycling performance for β-Sn and the type VIII clathrate electrodes is compared. Figure 7 shows the voltage profiles, capacity, and Coulombic efficiency (CE) for the two materials for 10 cycles. For β-Sn, the cycling performance is quite poor with a first cycle CE of 55% and low capacity retention, with loss of almost all of the initial capacity by the third cycle. In contrast, the Ba_8_Ga_15_Sn_31_ clathrate shows much better cycling and CE over the 10 cycles. For the clathrate electrode, the capacity begins at 670 mAh/g and decreases to 75 mAh/g after the 30th cycle, while the CE begins at 75% and then stabilized greater than 95% for the remaining cycles (Figure S10). While detailed investigation of electrolyte reduction processes on the type VIII clathrate surface is out of scope for this work, low CE and insufficiently passivating solid electrolyte interphases (SEI) have been observed before in type I silicon clathrate electrodes^12,46^ and may also be the case for the Sn clathrate. We expect, however, that optimization of the electrolyte and electrode construction will allow these materials to have better cycling performance to be competitive with other alloying anode materials.

The voltage profile for the first cycle of β-Sn (Figure 7c) shows the characteristic plateaus described by crystalline phase transformations during lithiation, as described before. However, during the first delithiation, the voltage profile shows a more sloped profile, as well as a lower capacity, resulting in a low CE. Based on the low capacity in subsequent cycles, we presume that the capacity fade originates from electronic disconnection of the β-Sn particles from the current collector due to volume contraction of the Li–Sn compounds during the first delithiation. Previously, an *in operando* NMR study showed that the capacity fade of Sn nanoparticles originated from the disconnection of Li_7_Sn_3_ during delithiation;^36^ we expect this effect to be exacerbated in our case due to the use of larger-sized Sn particles. In contrast, the type VIII clathrate shows a more reversible voltage profile with much better cycling stability, despite the larger initial particle size (Figure S2). The voltage profile of the first delithiation shows a gradually sloped profile from 0.01 to 0.60 V *vs* Li/Li^+^ with a much higher CE of 75%. In the second cycle, the lithiation curve shows a sloped profile ranging from 0.45 to 0.01 V *vs* Li/Li^+^ cutoff that mirrors the delithiation profile. In subsequent cycles, the electrode appears to be following the same reaction path due to the similar shape of the voltage profiles, suggesting that after the amorphization of the clathrate in the first cycle, the resulting amorphous phase cycles reversibly. There is still notable capacity fade during electrochemical cycling of the clathrate electrode, which could be due to volume expansion or unstable SEI formation.

Both electrodes were prepared using carbon black and poly(vinylidene difluoride) (PVDF) binder with slightly different active material ratios (80% for Ba_8_Ga_15_Sn_31_ and 90% for β-Sn). Considering the relatively similar electrode construction, the difference in cycling behavior is attributed to differences in the properties of the β-Sn and clathrate-active materials. We attribute the better electrochemical performance of the clathrate phase to the amorphous structural transformation. After the Ba_8_Ga_15_Sn_31_ is converted into an amorphous lithiated phase in the first cycle, subsequent delithiation and lithiation cycles go through solely amorphous solid solutions, which are known to reduce the stress^47−49^ experienced by the electrode during lithiation and delithiation, resulting in higher cycling stabilities. Considering the differences in particle sizes and active material ratios, future cycling studies will be needed to fully establish the benefit of the clathrate amorphous phase transformation on the cycling behavior.

### 3.6 Density Functional Theory (DFT) Calculations

**Figure 8:**
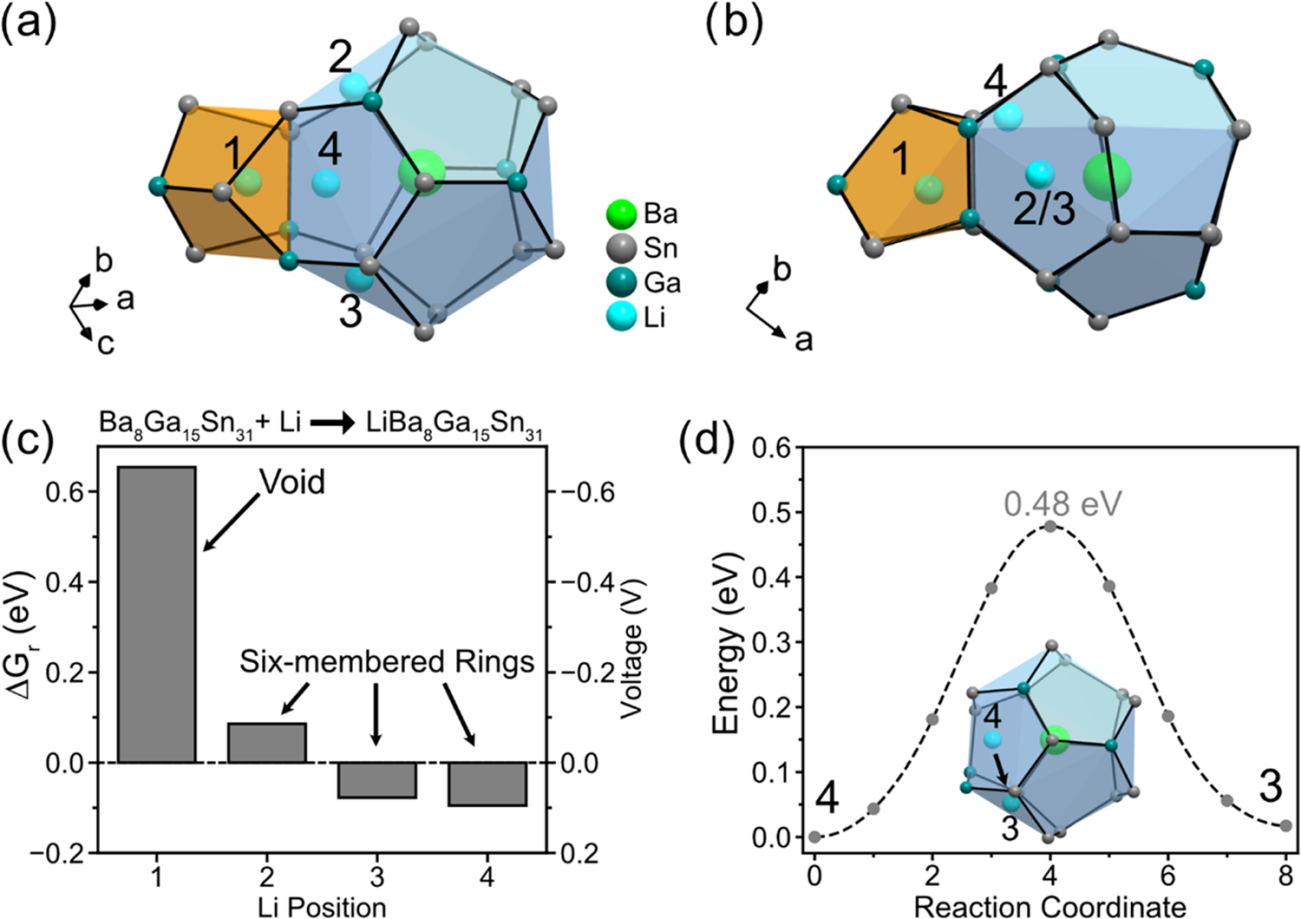
(a, b) Schematics of a void (orange) connected to distorted dodecahedra (blue) in the calculated type VIII Ba_8_Ga_15_Sn_31_ structure with investigated Li positions viewed in two perpendicular directions and labeled 1–4. (c) Gibbs free energy of reaction (Δ*G*_r_) for the reaction Ba_8_Ga_15_Sn_31_ + Li → LiBa_8_Ga_15_Sn_31_ for each different Li position. The corresponding potential (V *vs* Li/Li^+^) is shown on the right axis. (d) NEB-calculated minimum energy paths for migration of Li (cyan) between positions 4 and 3 in the distorted dodecahedra.

The aforementioned electrochemical and structural analyses showed that the lithiation process of the type VIII clathrate could be described as a conversion/alloying reaction to an amorphous phase. To assess the possibility of Li insertion into the type VIII Ba_8_Ga_15_Sn_31_ structure prior to electrochemical amorphization, we performed DFT calculations to evaluate the Li site energies and migration barriers between possible Li positions. The type VIII structure is composed of distorted dodecahedra that feature six-membered rings, which are potentially favorable structural features for Li insertion based on our previous calculations and experiments,^13,16,18,24^ as well as voids, which could potentially serve as access points for Li to enter the larger polyhedra. To evaluate the energetic favorability of Li insertion, the Gibbs free energy of reaction (Δ*G*_r_) was calculated for several Li positions where a negative Δ*G*_r_ represents a favorable reaction relative to the unlithiated Ba_8_Ga_15_Sn_31_ and Li metal. The pristine clathrate was calculated using the experimental type VIII Ba_8_Ga_15_Sn_31_ unit cell; the Ga substitutions on Sn sites were determined by the occupancy fraction derived from the single-crystal refinement while minimizing Ga–Ga bonds. The resulting structure has a lattice parameter of 11.838 Å (experimental = 11.589 Å) and a formation energy of −0.179 eV/atom. The lattice parameter is in good agreement with our other calculations for tetrel clathrates with the Perdew–Burke–Ernzerhof (PBE) functional, which show a value of around 0.2 Å larger relative to the experimental compound.^18^ The hypothetical type VIII Ba_8_Sn_46_ structure was also computed and resulted in a much higher formation energy of −0.051 eV/atom, demonstrating how the framework substitution of Ga stabilizes the structure. Then, single Li atoms were placed in the Ba_8_Ga_15_Sn_31_ lattice and relaxed to find the local minimum positions for LiBa_8_Ga_15_Sn_31_. Four Li positions were determined as local minima based on this procedure and are overlaid on the partial structure of the pristine clathrate in Figure 8a,b, which displays a void (shaded orange) connected to the distorted dodecahedra (shaded light blue).

Li position 1 represents Li in the center of the void that forms in between the distorted dodecahedra (as shown more clearly in Figures 1b and 2a). The Gibbs free energy of reaction (Δ*G*_r_) of this position is shown in Figure 8c as Li position “1” with a value of 0.62 eV, suggesting that this position does not result in a favorable reaction. Observation of the Li distances between the unrelaxed (Li–Sn/Ga = 2.22 Å) and relaxed structures (Li–Sn/Ga = 2.54 Å) suggests that the high energy originates from the expansion of the void to maintain acceptable Li–Sn/Ga distances, resulting in significant disturbance of the surrounding Sn/Ge framework. Due to the positive Δ*G*_r_ of this position, we conclude that the voids in the type VIII structure are too small to be occupied by Li.

In our previous studies, we identified hexagonal faces as important features for Li to coordinate to or migrate through in clathrate structures.^16,24^ In particular, for the guest-free, type I Sn clathrate (Sn_46_), the center of the hexagonal face was determined as a favorable Li position. Due to the large size of the hexagon composed of Sn atoms,^24^ appropriate Li–Sn distances (2.7–2.8 Å) can be maintained. Therefore, we chose to investigate the Li positions in the center of the Sn/Ga six-membered rings in the distorted dodecahedra in the type VIII structure. The energies of the six-membered ring positions are represented by positions 2 and 3; the two positions are distinct because of the different positioning of the adjacent Ga atoms in the six-membered ring (*i.e*., position 2 has two Ga atoms on opposite sides of the six-membered ring, while position 3 has two Ga bonded together next to the void). The Δ*G*_r_ for Li in these positions are +0.06 and −0.087 eV, respectively, suggesting that the local positions of the Ga atoms in the six-membered ring affect the Li site energy. These energies are lower than if Li was placed in the void (position 1) but higher than Li insertion into the Sn_46_ hexagon (−0.30 eV).^24^ After relaxation, there is a slight rearrangement of the six-membered ring and the Ba atom in the adjacent cage is perturbed from its original position, suggesting that these disturbances might be the origin of the higher relative energy. Position 4 is also close to a six-membered ring, but this six-membered ring is distorted because part of it forms the void. Similar to position 3, this position has a Gibbs free energy of reaction (Δ*G*_r_) of −0.093 eV, suggesting that the six-membered rings have similar energies for Li insertion. The lattice parameters of the structures after Li insertion are presented in Table S7, which show that relative to the unlithiated structure, the lattice parameter increased by ∼0.04–0.05 Å.

Next, climbing image nudged elastic band (NEB) calculations were used to estimate the Li migration barrier between adjacent Li positions within the distorted dodecahedra. The NEB energy reaction path between positions 4 and 3 is presented in Figure 8d, with the inset showing the beginning and ending Li positions in the cage. The pathway for the migration involves Li moving along the side of the cage, while the Ba atom in the cage moves slightly to maintain its distance from the migrating Li atom. This pathway results in a barrier of 0.48 eV, which is reasonable for room-temperature diffusion,^50^ suggesting that Li migration could be feasible within the structure between the six-membered rings of the distorted dodecahedra.

The DFT results suggest that Li insertion is energetically feasible as it predicts Li insertion positions with negative Gibbs free energy (positive voltage *vs* Li/Li^+^) with a reasonable migration barrier (0.48 eV) for bulk diffusion. However, whether this process is seen experimentally depends on competing reactions that can occur in a similar voltage range. Since the potential for the alloying reaction of Ba_8_Ga_15_Sn_31_ to form the Li-rich amorphous phase is at 0.25 V *vs* Li/Li^+^ (Figure 3), we do not expect Li insertion into the clathrate lattice to occur since alloying occurs at a higher reaction voltage compared to the insertion reactions (*e.g*., Li insertion into position 3 or 4 at ∼0.10 V *vs* Li/Li^+^ per Figure 8c) as the Li content increases. This is further supported by the lack of significant change in the clathrate lattice parameter before and after lithiation, as determined from the PDF refinement (Tables S3 and S6). We attribute the relatively high Li site energies in the Ba_8_Ga_15_Sn_31_ structure to the presence of the Ba guest atoms since our previous calculations showed that the guest-free Sn clathrate (Sn_46_) has a favorable Li site energy of −0.30 eV. To accommodate the Li atom, the Ba atoms move from their favorable positions in the center of the dodecahedral cages, which incurs an energetic cost to the system and results in a higher-energy Li position compared to that if the Ba was not present. In addition, the Ba and Li atoms are expected to have positive charges, which would repulse and increase the energy of the position. If guest-free type VIII structures could be obtained, they would be particularly interesting for insertion electrodes due to a large amount of six-membered rings in the polyhedra. Unfortunately, there have been few reports of tetrel clathrates forming with the type VIII structure, all of which have a full guest atom occupancy and form with framework substitutions that necessitate guest atoms as electron donors.^22,51−53^

## 4 Discussion

### 4.1 Comparison of the Lithiation Mechanisms of Ba_8_Ge_43_ and Ba_8_Ga_15_Sn_31_

**Figure 9:**
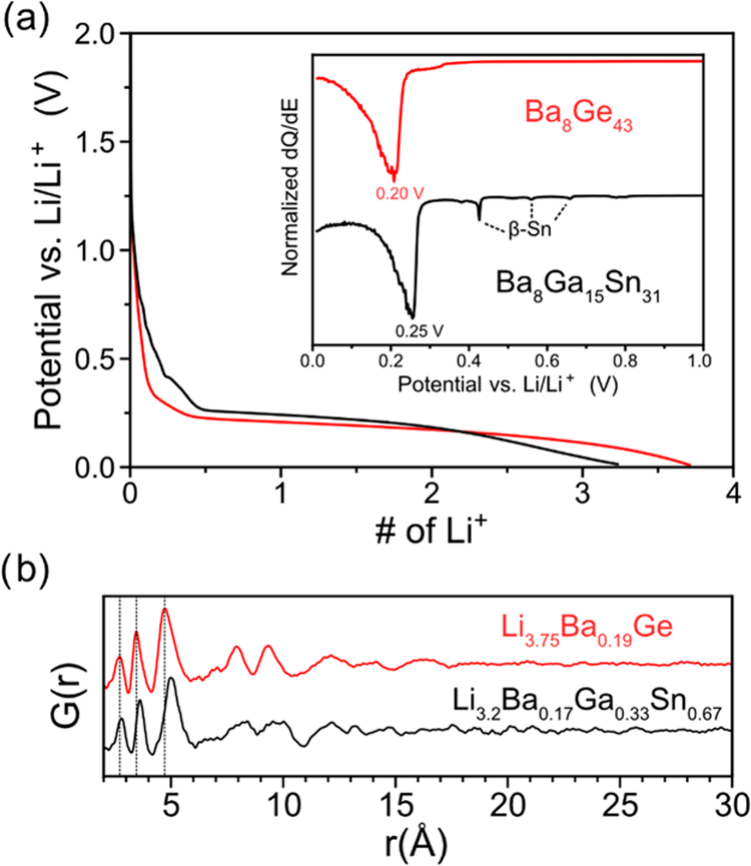
(a) Voltage profile for lithiation of Ba_8_Ge_43_ and Ba_8_Ga_15_Sn_31_ and corresponding d*Q*/d*E* plots. (b) Comparison of the PDFs from the most lithiated phases of the Ba_8_Ge_43_ clathrate and the Ba_8_Ga_15_Sn_31_ clathrate. The compositions are normalized to the amount of nonalkali/alkaline earth metal atoms. The data for Ba_8_Ge_43_ are reproduced from ref (19).

The structural transformation of the type VIII clathrate Ba_8_Ga_15_Sn_31_ during lithiation is very similar to those observed in our study on type I Ge clathrates.^19^ To illustrate this, the voltage profiles, d*Q*/d*E* plots, and PDFs of the fully lithiated structures of Ba_8_Ga_15_Sn_31_ and Ba_8_Ge_43_ are presented in Figure 9. Both clathrate voltage profiles show a plateau (seen as a large peak in the d*Q*/d*E* plot) followed by a sloped region until the end of lithiation. This shape of voltage profile suggests that upon lithiation, the clathrate phase undergoes a two-phase reaction to form a lithiated amorphous phase, which is further lithiated *via* a solid-solution mechanism. The PDFs of the fully lithiated amorphous phases derived from Ba_8_Ge_43_ and Ba_8_Ga_15_Sn_31_ show correlations with similar positions up to 15 Å, with those in Li_3.2_Ba_0.17_Ga_0.33_Sn_0.67_ shifted to slightly higher *r* values due to the longer bond lengths of Ga and Sn atoms.

**Figure 10:**
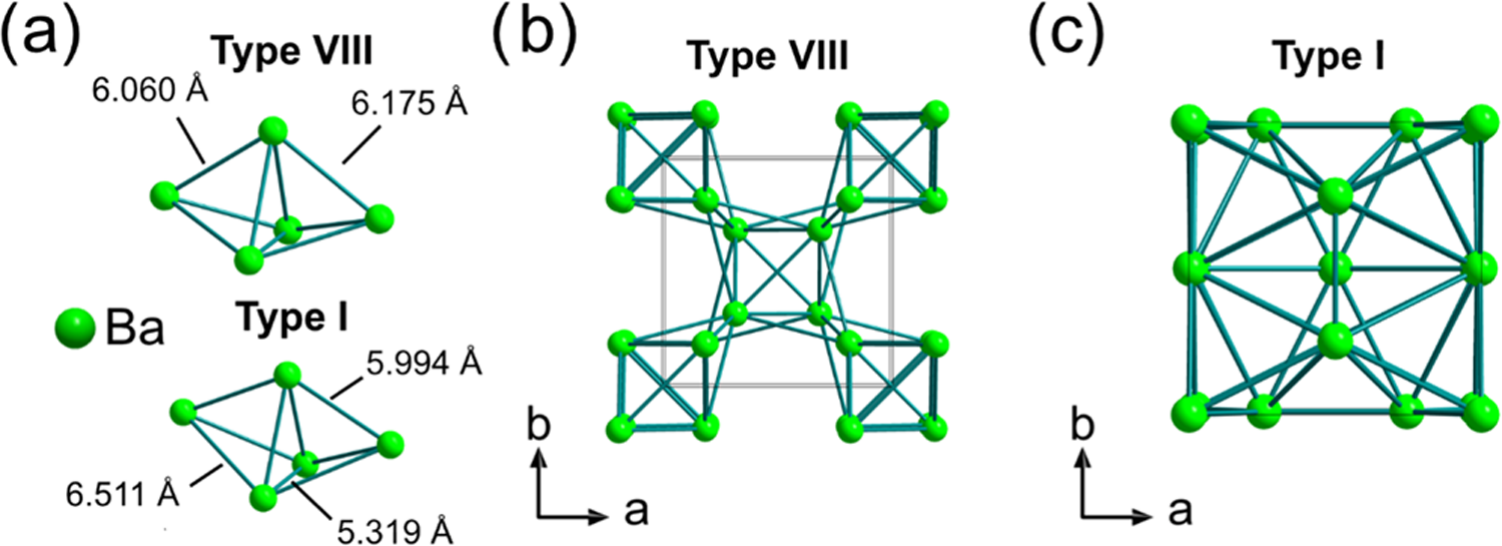
(a) Schematic of the common local structure features in the type VIII Ba_8_Ga_15_Sn_31_ and type I Ba_8_Ge_43_ clathrate Ba sublattices. Ba sublattice of the (b) type VIII Ba_8_Ga_15_Sn_31_ clathrate and (c) type I Ba_8_Ge_43_ clathrate.

The close similarity of the structures and reaction mechanisms for Ba_8_Ge_43_ and Ba_8_Ga_15_Sn_31_ during lithiation reveals several important characteristics for the lithiation of Ba-filled clathrates. First, the composition and local structure of the clathrate seem to be more important for the Li alloying reaction rather than the initial crystal structure. While the Ba_8_Ge_43_ clathrate is described by the type I and Ba_8_Ga_15_Sn_31_ by the type VIII structure, the spacing of Ba atoms and the local structures are relatively similar. This is illustrated in Figure 10, which shows a schematic of the local structure around Ba atoms that is common to both clathrate structure types with select Ba–Ba distances labeled (Figure 10a) and a unit cell of the Ba sublattice in the type VIII (Figure 10b) and type I (Figure 10c) clathrate structures. The common local feature of the Ba sublattice comprises two face-sharing tetrahedra, which then combine in face-sharing and corner-sharing arrangements in the type VIII structure and in face-sharing arrangements for the type I structure. Both Ba sublattices have similar Ba–Ba distances of around 6 Å, but the distances in Ba_8_Ge_43_ show a wider spread as a result of the different Ba sites in the type I structure (corresponding to the centers of the Ge_20_ and Ge_24_ cages). When the clathrate structure is eventually converted to an amorphous phase *via* electrochemical lithiation, the similar local structuring of Ba within the framework structure has a larger effect on the structures of the resulting amorphous phases compared to the small differences in the arrangement of framework/guest atoms in the original clathrate structure.

Another important consideration is that despite differences in composition, both clathrates react through a similar mechanism, as supported by the similarly shaped voltage curves and PDFs after lithiation (Figure 9). The PDF of the framework-substituted, type I clathrate Ba_8_Al_16_Ge_30_ also displayed similar features to that for Ba_8_Ge_43_^19^ and Ba_8_Ga_15_Sn_31_ after lithiation. The shared reaction mechanism between the Ba-filled clathrates with different compositions is interesting as elemental Ge,^20^ Sn,^32^ Al,^54^ and Ga^55^ all have distinctly different lithiation pathways at room temperature and involve crystallization to Li binary phases. Furthermore, alloys of Sn, such as SnSb^56,57^ and GaSn,^58^ show electrochemical features during lithiation that can be traced back to the lithiation of the constituent elemental phases, suggesting that significant demixing and crystallization occur during the lithiation process despite the alloyed state of the starting material. We attribute the distinct electrochemical properties of the clathrates to the unique structure where Ba guest atoms and framework atoms form an atomically mixed amorphous phase after lithiation, which kinetically prevents demixing of the constituent elements.

The origin of this amorphous pathway in the lithiation of type VIII clathrate can be attributed to kinetic limitations of the lithiation of the host structure. The Ba atoms are presumed to act as “pillars”, which serve to break up long-range order and prevent the bulk crystallization of Li–Sn/Ga phases. This is similar to what we observed in the Ba_8_Ge_43_ system,^19^ but the lack of known binary Ba–Sn clathrate phases (*i.e*., Ba_8_Sn_43_) precludes a direct comparison to Ba_8_Ge_43_. In contrast, β-Sn undergoes phase transformations at low Li content to form crystalline phases (*e.g*., Li_2_Sn_5_, LiSn) that follow the binary phase diagram. Comparison of the crystal structures of β-Sn, Li_2_Sn_5_, and LiSn (Figure S11) shows a common square Sn unit that is progressively broken up as the Li content increases. It has been suggested that this common feature in the Li–Sn crystalline phases enables kinetically facile phase transformations with good reversibility.^33,35^ In the case of the clathrate, the original crystal structure is unrelated to Li–Sn or Li–Ga binary phases, which means that a significant amount of rearrangement and demixing of Ba/Sn/Ga would be required to form crystalline binary phases, a process that is highly unlikely at room temperature. As a result of these kinetic limitations, the phases that form are amorphous with the mobile Li ions driving the structural changes.

The amorphous lithiation pathway of Ba_8_Ga_15_Sn_31_ has significant effects on the voltage profile relative to that in β-Sn. Most notably, the voltage of reaction with lithium is lower for the clathrate (0.25 V *vs* Li/Li^+^) than for β-Sn (0.45 V), which is consistent with the formation of a higher-energy amorphous phase. In addition, the presence of Ba and Ga substitutions in the clathrate is expected to further increase the Gibbs free energy of the lithiated phase (*i.e*., lower the voltage) as seen in our previous study on the Ba_8_Al_*y*_Ge_46–*y*_ (0 < *y* < 16) clathrates, which demonstrated that increasing Al substitution decreased the reaction potential compared to Ba_8_Ge_43_.^18^ We speculate that Ga substitution could have a similar effect on the reaction potential. We also note that the clathrate reacts with a lower amount of Li compared to β-Sn (3.2 Li for the clathrate, 3.4 Li for β-Sn), which was also a notable effect when increasing the degree of Al substitution in Ba_8_Al_*y*_Ge_46–*y*_.

After the initial conversion of the clathrate to an amorphous phase in the first cycle, the delithiation profile is sloped, showing no distinct plateaus corresponding to phase transformations. This sloped behavior suggests that delithiation of the Li_*x*_Ba_0.17_Ga_0.33_Sn_0.67_ phase goes through a solid-solution mechanism where the Li content varies continuously during the reaction. After one lithiation and delithiation cycle, the originally crystalline clathrate is suspected to now be amorphous, similar to what occurs for diamond cubic Si.^47,48^ From the PDF analysis of Ba_8_Ge_43_ after full lithiation and delithiation, the local structure was found to be similar to that of the original pristine crystalline clathrate but did not exhibit long-range order. On the basis of the similar reaction mechanisms we have observed in the Ba-containing clathrates, we expect delithiated Li_x_Ba_0.17_Ga_0.33_Sn_0.67_ to have a similarly structured amorphous phase. During cycling, the shape of the voltage profile does not change significantly, suggesting that the amorphous phase formed in the first cycle is able to be reversibly cycled. However, future PDF characterization after cycling or *in situ* measurements will be needed to confirm this.

### 4.2 Design Strategies for Li-Ion Battery Applications

Given that Ba_8_Ge_43_, Ba_8_Al_16_Ge_30_, and Ba_8_Ga_15_Sn_31_ all undergo amorphous phase transformations upon reaction with Li, we presume that this amorphous reaction mechanism might be a general feature of Li alloying with guest-filled clathrates. An exception to this trend is the type I clathrate Ba_8_Al_16_Si_30_, which we found to display electrochemical reactions dominated by surface reactions rather than bulk alloying reactions.^12,15^ Notably, the presence of guest atoms is important as clathrates with type II structures that can be synthesized without guest atoms (*i.e*. vacant cages) can go through topotactic insertion reactions without transforming to amorphous phases at low degrees of lithiation. This has been observed in Si_136_^9,16^ and also likely occurs in Ge_136_,^17^ but amorphous phase transformations are observed if the type II clathrates are prepared with guest atoms^11^ or overlithiated.^16^ On the basis of the DFT calculations presented herein (Figure 8) and our previous calculations, we conclude that clathrates with occupied cages will favor amorphous phase transformations over insertion reactions since the energies for the Li sites (for insertion processes) and migration barriers increase if the cages are already occupied.^13,18,24^ By assuming a common amorphous reaction mechanism among clathrates containing guest atoms, we can propose a general strategy for designing clathrates to serve as alloying anodes, which could be helpful in directing future research. First, it is important to emphasize that the initial crystalline clathrate structure will be converted into an amorphous phase in the first lithiation process and later cycles will involve lithiation/delithiation of amorphous phases. This is similar to the electrochemical reaction of Li with diamond cubic Si, where the diamond structure is not recovered after Li is removed.^47,48,59^ Next, the guest atoms (*i.e*., Ba) in the clathrate structure serve as electrochemically inactive but structurally important components, with the role of kinetically frustrating the formation of crystalline phases. Suppressing the formation of crystalline phases mitigates large changes in volume, which can lead to better cycling retention. Then, substitution of elements on tetrel framework sites (*i.e*., with Ga or Al) allows for tuning of the reaction voltage and lithiation capacity of the amorphous phase.

In terms of the guest atom selection, the optimal choice is the lightest atom possible to maintain a high gravimetric specific capacity while still having the benefit of serving as “pillar” to suppress crystalline phase transformations. In this regard, the lightest reported guest atom (other than Li) for clathrates is Na.^1^ However, our previous studies investigating the properties of Na-filled Si clathrates in batteries suggest that the Na guest atom does not suppress crystalline phase transformations, as the formation of crystalline Li_15_Si_4_ was observed after full lithiation of both type I (Na_8_Si_46_) and type II (Na_24_Si_136_) clathrates.^11,14^ The lithiation of Na-filled Ge clathrates (*e.g.*, Na_24–*x*_Ge_136_,^5,60^ Na_8_Zn_4_Ge_42_,^61^ Na_8_Ga_8_Ge_38_^62^) has yet to be reported but would be promising if phase transformations were disrupted. However, we think this is unlikely as Ge generally shows a higher propensity than Si to undergo crystalline phase transformations during lithiation, at least when adopting the diamond cubic structure. The next lightest guest atom for clathrates is K, with many K-filled Si, Ge, and Sn clathrates reported.^63−66^ We believe that K-filled clathrates are particularly promising due to the significantly larger atomic size of K compared to that of Na, which might affect the room-temperature lithiation pathway significantly while still having a relatively low atomic weight. We have preliminary data of the K-containing Ge clathrate, K_8_Li*_x_*Ge_46–*x*_,^67^ showing that the voltage profile displays a single major plateau during lithiation and then a sloped delithiation curve (Figure S12a,b), similar to Ba_8_Ge_43_ and Ba_8_Ga_15_Sn_31_, suggesting that it might also go through an amorphous phase transformation. PXRD after full lithiation showed that the pristine clathrate reflections decreased in intensity without any other phases being detected, further supporting an amorphous phase transformation (Figure S12c). Due to the large particle sizes of the K_8_Li*_x_*Ge_46–*x*_ (Figure S12d,e), we suspect that there could be kinetic limitations during the lithiation, which resulted in incomplete conversion of the clathrate. Further structural characterization with PDF analysis of the lithiation intermediates of K_8_Li*_x_*Ge_46–*x*_ will be needed to confirm the suspected amorphous phase transformation. Tetrel clathrates have also been reported with Rb, Cs, Ca, Sr, and rare-earth guest atoms;^1,68−72^ however, these guest atoms are heavier than K and Na and would be less attractive in terms of gravimetric capacity. More work examining the effect of the size and valency of the guest atom on the subsequent lithiation pathways would be warranted for assessing the viability of heavier guest atoms.

Substitution of framework atoms with other elements is also an interesting design avenue as it allows for tuning of the reaction capacity/voltage while also being able to compensate for the extra mass originating from the guest atoms. In the case of Ba_8_Ga_15_Sn_31_, the molar mass (5829.3 g/mol) is lower than that of pure Sn (6409.8 g/mol) on a per-atom basis, meaning that the additional mass from Ba atoms is negated by the incorporation of the Ga. This means that despite the lower amount of Li reacting with Ba_8_Ga_15_Sn_31_ (3.2 Li per Sn/Ga *vs* 3.4 Li per Sn for β-Sn as seen in our results), both Ba_8_Ga_15_Sn_31_ and β-Sn have similar initial capacities (∼700 mAh/g). In addition to the modification of the capacity in terms of weight or amount of reacted Li, our findings suggest that adding substitutions decreases the reaction voltage (also seen when substituting Al for Ge in Ba_8_Al_*y*_Ge_46–*y*_),^18,19^ which would result in a higher overall cell voltage when the clathrate is coupled with a cathode in a full cell, resulting in a higher energy density. The effect of substitutions on the capacity, reaction voltage, and amount of Li that can react with the electrode means that there should be an optimal point at which the energy density is maximized. Considering the K-filled Ge and Sn clathrates, Al-substituted clathrates such as K_8_Al_8_Ge_38_ and K_8_Al_8_Sn_38_^73^ are particularly promising due to the low weight of Al, which could nullify the weight increase from K while also potentially decreasing the reaction voltage with minimal decreases to the amount of Li that reacts. If these clathrates go through amorphous lithiation pathways like the Ba clathrates, then they could be promising alternatives to elemental Ge and Sn anodes for Li-ion batteries.

## 5 Conclusions

In this work, the lithiation pathway of the type VIII Ba_8_Ga_15_Sn_31_ clathrate is investigated with electrochemical analysis and total scattering X-ray powder diffraction measurements. Ba_8_Ga_15_Sn_31_ and β-Sn (as a comparison) are lithiated to similar amounts and then subjected to X-ray characterization. The lithiation voltage profile for the type VIII clathrate shows a single plateau at 0.25 V followed by a sloping profile, while the voltage profile of β-Sn has three plateaus corresponding to known phase transformations to Li–Sn crystalline phases. Structure function patterns at different points in lithiation confirm that β-Sn goes through crystalline phase transformations, while the type VIII clathrate undergoes an amorphous phase transition. PDF analysis confirms the phase transformation of the type VIII Ba_8_Ga_15_Sn_31_ clathrate to a highly lithiated amorphous phase that lacks long-range order. The PDF analysis also suggests that the amorphous Li_*x*_Ba_0.17_Ga_0.33_Sn_0.67_ phases share similar local structures to those in crystalline Li–Sn phases. Galvanostatic cycling experiments showed that the type VIII clathrate resulted in better cycling and Coulombic efficiency than β-Sn, which is attributed to reduced stresses and deleterious effects of volume expansion/contraction during cycling on account of the solid-solution lithiation/delithiation mechanism in the clathrate electrode. DFT calculations to assess the possibility of topotactic Li insertion into the Ba_8_Ga_15_Sn_31_ clathrate suggest that the Li insertion site energy is too high to be competitive with the amorphous alloying reaction due to the presence of the Ba guest atoms in the cages.

Overall, we find that the lithiation of the type VIII Ba_8_Ga_15_Sn_31_ is very similar to that observed in the type I Ba_8_Ge_43_ clathrate investigated in our previous work. The similar reaction mechanism and structure of the amorphous phases are attributed to the unique clathrate structure in which Ba atoms are distributed through the cage framework and act as “pillars” to prevent long-range ordering. The resulting amorphous phase has distinct electrochemical reactions with Li relative to those in the elemental phases and potentially beneficial electrochemical properties such as a lower reaction voltage and better cycling stability due to the solid-solution mechanism. Tetrel clathrates represent a wide design space for tuning the material properties because of the numerous types of guest atoms, structures, and compositions. Based on the results presented here, we expect that many other clathrate compositions could undergo amorphous alloying reactions with novel and tunable electrochemical properties for Li-ion battery applications.

## Supporting Information Available

Detailed experimental procedures for the synthesis of type VIII clathrate, electrochemical measurements, sample preparation for synchrotron measurements, pair distribution function analysis, powder X-ray diffraction and scanning electron microscopy, and density functional theory calculations; supporting tables include crystallographic data for Ba_8_Ga_14.9_Sn_31.1_ from single-crystal XRD; PDFgui refinement parameters for pristine Ba_8_Ga_15_Sn_31_ and pristine β-Sn, and Li*_x_*Sn and Li*_x_*Ba_0.17_Ga_0.33_Sn_0.67_ samples fit to different phase combinations; the measured voltages and capacity for each sample after electrochemical lithiation; and lattice constants from the DFT-calculated structures (LiBa_8_Ga_15_Sn_31_); supporting figures include SEM images and EDS of two Ba_8_Ga_15_Sn_31_ single-crystal particles; PXRD of the hand-ground type VIII crystals; SEM images of Ba_8_Ga_15_Sn_31_ and β-Sn electrodes prior to electrochemical lithiation; calculated total and partial PDF patterns for LiSn, Li_7_Sn_3_, Li_7_Sn_2_, and type VIII clathrate Ba_8_Ga_15_Sn_31_; crystal model schematics of β-Sn, LiSn, Li_2_Sn_5_, and Li_7_Sn_3_; PDF refinements for pristine β-Sn, lithiated β-Sn (Li*_x_*Sn) samples, and lithiated Ba_8_Ga_15_Sn_31_ (Li*_x_*Ba_0.17_Ga_0.33_Sn_0.67_) samples; variable temperature PDF during *in situ* heating from 310 to 420 K for Li_3.2_Ba_0.17_Ga_0.33_Sn_0.67_; capacity and Coulombic efficiency *vs* cycle number for β-Sn and the type VIII Ba_8_Ga_15_Sn_31_ clathrate for 30 cycles; and voltage profile, d*Q*/d*E* plot, PXRD, and SEM of type I K_8_Li*_x_*Ge_44*–x*_ clathrate electrode before and after full lithiation (PDF)
CIF for the single-crystal XRD refinement of the as-synthesized Ba_8_Ga_15_Sn_31_ (CIF)
CIF for the DFT-calculated structure of Ba_8_Ga_15_Sn_31_ (CIF)

## Supplementary Material

am1c07240_si_001.cif

am1c07240_si_002.cif
