# Supplementary material for: Structural and Electrochemical Properties of Type VIII Ba8Ga16−δSn30+δ Clathrate (δ ≈ 1) during Lithiation
Source: ACS Appl Mater Interfaces. 2021 Sep 3;13(36):42564–78. doi: 10.1021/acsami.1c07240 (PMC8447186; doi:10.1021/acsami.1c07240)
Supplement: Supplementary file 3 — am1c07240_si_003.pdf [file am1c07240_si_003.pdf]

Supporting Information for:

Structural and Electrochemical Properties of Type VIII $Ba_8Ga_{16-\delta}Sn_{30+\delta}$ Clathrate ($\delta \approx 1$) during Lithiation

*Andrew Dopilka[1], Amanda Childs[2], Alexander Ovchinnikov[2,3], Ran Zhao[4], Svilen Bobev[2], Xihong Peng[5], Candace K. Chan[1,6]**

1. Materials Science and Engineering, School for Engineering of Matter, Transport and Energy, Arizona State University, P.O. Box 876106, Tempe, AZ 85827
2. Department of Chemistry and Biochemistry, University of Delaware, Newark, DE 19716
3. Department of Materials and Environmental Chemistry, Stockholm University, Svante Arrhenius väg 16 C, 10691 Stockholm, Sweden
4. School of Molecular Sciences, Arizona State University, P.O. Box 871604, Tempe, Arizona 85287, United States
5. College of Integrative Sciences and Arts, Arizona State University Polytechnic Campus, Mesa, AZ, 85212
6. Department of Heterogenous Catalysis, Max-Planck-Institut für Kohlenforschung, Kaiser-Wilhelm-Platz 1, 45470 Mülheim an der Ruhr, Germany

Table of Contents

1. Experimental Procedures

1.1 Synthesis of Type VIII Clathrate $Ba_8Ga_{15}Sn_{31}$
1.2 Single Crystal X-ray Diffraction
1.3 Electrochemical Measurements
1.4 Pair Distribution Function Analysis
1.5 Powder X-ray Diffraction
1.6 Scanning Electron Microscopy
1.7 Density Functional Theory (DFT) Calculations
1.8 Synthesis and Characterization of Type I Clathrate $K_8Li_xGe_{46-x}$

2. Supporting Tables

Table S1. Crystallographic data for $Ba_8Ga_{14.9}Sn_{31.1}$ from single crystal XRD
Table S2. Atomic coordinates, occupancies, and equivalent isotropic displacement parameters (U_{eq}) of $Ba_8Ga_{14.9}Sn_{31.1}$ from single crystal XRD refinement
Table S3. PDFgui refinement parameters for pristine $Ba_8Ga_{15}Sn_{31}$.
Table S4. The measured voltages and capacity for each sample after electrochemical lithiation.
Table S5. PDFgui refinement parameters for pristine β-Sn and Li_xSn samples fit to different phase combinations.
Table S6. PDFgui refinement parameters for $Li_xBa_{0.17}Ga_{0.33}Sn_{0.67}$ samples fit to different phase combinations.
Table S7. Lattice constants from the DFT calculated structures ($LiBa_8Ga_{15}Sn_{31}$).

3. Supporting Figures

Figure S1. SEM images and EDS spectra of two $Ba_8Ga_{15}Sn_{31}$ single crystal particles and PXRD of the type VIII clathrate powder.
Figure S2. SEM images of $Ba_8Ga_{15}Sn_{31}$ and β-Sn electrodes prior to electrochemical lithiation.
Figure S3. Calculated total and partial PDF patterns for LiSn, Li_7Sn_3, Li_7Sn_2. Crystal model schematics of LiSn and Li_7Sn_3.
Figure S4. Calculated total and partial PDF pattern for type VIII clathrate $Ba_8Ga_{15}Sn_{31}$.
Figure S5. PDF refinement and crystal structure of pristine β-Sn.
Figure S6. PDF refinements for lithiated β-Sn (Li_xSn) samples.
Figure S7. PDF refinements for lithiated $Ba_8Ga_{15}Sn_{31}$ ($Li_xBa_{0.17}Ga_{0.33}Sn_{0.67}$) samples.
Figure S8. Crystal model view of Li_7Sn_3 showing only Sn atoms to demonstrate certain Sn-Sn distances.
Figure S9. Variable temperature PDF during *in situ* heating from 310 – 420 K for $Li_{3.2}Ba_{0.17}Ga_{0.33}Sn_{0.67}$
Figure S10. Capacity and Coulombic efficiency vs cycle number for β-Sn and the type VIII $Ba_8Ga_{15}Sn_{31}$ clathrate for 30 cycles.
Figure S11. Comparison of the crystal structures of β-Sn, Li_2Sn_5, and LiSn, which show a common square Sn unit.
Figure S12. Voltage profile, dQ/dE plot, PXRD and SEM of electrodes before and after full lithiation for the type I $K_8Li_xGe_{44-x}$ clathrate electrode.

1. Experimental Procedures

1.1. Synthesis of Type VIII Clathrate $Ba_8Ga_{15}Sn_{31}$

The synthesis was done in a manner, similar to a previous work.[1] Large crystals of the type VIII clathrate with refined composition $Ba_8Ga_{\sim15}Sn_{\sim31}$ were grown by Ga/Sn flux reaction. Elemental Ba, Ga, and Sn (commercial grade materials with stated purity 99.9% wt or greater) were combined in a 8:40:65 molar ratio to a 2 cm^3 alumina crucible that was subsequently flamed-sealed in an evacuated fused silica tube. The sample was heated to 500 ºC (20 ºC/hr) then held at 500 ºC for 500 hrs. After cooling to 400 ºC (-5 ºC/hr), the Ga/Sn flux was removed by centrifugation. The large crystals were then hand ground prior to electrochemical testing.

1.2. Single Crystal X-ray Diffraction

Single-crystal X-ray diffraction measurements were performed on a Bruker APEX-II CCD diffractometer stocked with a sealed-tube Mo Kα ($\lambda = 0.71073$ Å) source. Proper single crystals were selected under an optical microscope, covered in Paratone-N oil and placed onto a holder loop made of low-background plastic. The holder was transported to the goniometer head, where a cold stream of nitrogen gas was used to harden the oil and to prevent oxidation/decomposition over the span of the data collection. The temperature was kept at 200(2) K throughout the experiment. Data acquisition was performed with Bruker-provided software. Measurements were collected in batch runs with a frame width of 0.8° in ω and θ. Data reduction and scaling were managed using SAINT.[2] Absorption correction was implemented using SADABS software program.[3] The structure was solved using ShelXT[4] in Olex2[5] and refined with the full-matrix least squares method on F^2, as implemented in SHELXL[6]. Atomic coordinates were standardized using STRUCTURE TIDY[7] and tables were generated using ReportPlus. The CIF from this refinement is available as a supporting information file.

1.3. Electrochemical Measurements

The clathrate powder was prepared into slurries by mixing the clathrate sample with 10 wt% carbon black (to serve as conducting additive) and 10 wt% polyvinylidene difluoride (PVDF) (to serve as

binder) in N-methyl pyrrolidone (NMP) as solvent. β-Sn powder (Sigma Aldrich, 10 microns, 99%) was used as-received and prepared with using 5 wt% carbon black and 5 wt% PVDF for comparison to the clathrate samples. The slurries were stirred overnight and coated onto Cu foil current collectors using a Meyer rod, followed by heating at 120 ^oC to remove the solvent.

For cycling experiments, the electrodes were evaluated in CR2302 coin cells with Li metal as the counter electrode, Celgard 2500 as separator, and 1 M $LiPF_6$ in EC:DMC:DEC, 4:3:3 by volume, with VC additive (LBC3051C, MTI) as the electrolyte. Electrochemical testing was performed using a Biologic VMP3 galvanostat/potentiostat. Galvanostatic measurements were performed from 0.01 – 2.5 V vs. Li/Li^+ at 12.5 mA/g or 25 mA/g.

For preparing lithiated samples for total scattering measurements, the composite electrodes were sealed in pouch cells made from aluminized polymer laminate rather than coin cells. After the cell was lithiated to the desired amount, it was allowed to relax at open circuit for 5 hours prior to disassembly. Two to three repeats of a single electrochemical experiment were conducted to provide enough lithiated powder for structural characterization. After the electrochemical lithiation was complete, the pouch cell was taken into an argon-filled glovebox and opened. The electrode was then immersed in 10 mL of dimethyl carbonate for 30 seconds to wash off any excess battery electrolyte. After the electrode was dry, the lithiated powder was scraped off the copper current collector using a knife. The extracted powder was then crushed in a mortar with a pestle to break up any agglomeration. The powder was sealed in a 2 mL centrifuge tube and then sealed under argon in a polyfoil bag before shipping to the synchrotron facility.

1.4. Pair Distribution Function (PDF) Analysis

Lithiated powders were loaded into 0.8 mm diameter borosilicate capillaries and sealed with wax and superglue inside an argon-filled glovebox, while pristine powders were loaded into the capillaries in ambient conditions. PDF measurements were performed at Diamond Light Source (Didcot, United Kingdom) at the I15-I dedicated PDF beamline with 76 keV X-rays (wavelength of 0.161669 Å) and 2D PerkinElmer image plate detectors. The detector geometry allowed collection of total scattering data to Q

= 30 Å^{-1}. *Ex situ* PDF measurements were carried out at room temperature. The *in situ* PDF heating measurements were carried out from 300 – 450 K (temperature interval of 10 K) using an Oxford Instruments Cryojet 5. After reaching each hold temperature, there was a 1.5 min equilibration period and then the scattering data were collected with a 5 minute collection time. The heating temperature was limited to 450 K to avoid decomposition of the PVDF binder in the composite electrodes.

PDFs were generated from the total scattering data using PDFgetx3[8] within the xPDFsuite software package,[9] wherein the measured total scattering intensities, $I(Q)$, are corrected to obtain the coherent scattering, $I_c(Q)$, and transformed into the structure function, $S(Q)$, according to equation 1,[8]

$$S(Q) = \frac{I_c(Q) - \langle f(Q)^2 \rangle + \langle f(Q) \rangle^2}{\langle f(Q) \rangle^2} \qquad (1)$$

where $f(Q)$ is the atomic scattering factor, which is averaged over all atom types in the sample. The PDF, $G(r)$, is obtained from the Fourier transform of $S(Q)$ as shown in equation 2:

$$G(r) = \frac{2}{\pi} \int_{Q_{min}}^{Q_{max}} Q[S(Q) - 1] \sin(Qr)\, dQ \qquad (2)$$

To generate the PDFs, the following parameters were used: Q_{min} = 0.5 Å^{-1}, Q_{max} = 25 Å,$^{-1}$ r_{step} = 0.1 Å, and r_{poly} = 0.9. The nominal composition for lithiated samples was obtained from the charge passed during the electrochemical measurements.

PDF refinements were carried out using PDFgui.[10] No attempts were made to consider the presence of binder, carbon black, or solid electrolyte interphase (SEI) components in the samples. Previous reports have shown that these components add negligible contribution to the PDF data.[11] PDF refinements were performed using Q_{damp} = 0.0247 and Q_{broad} = 0.0151 (obtained from refinement of a NIST Si standard). To refine a PDF pattern, the major phase of the pattern was first selected and then scale factor and lattice parameter were refined. Then the isotropic displacement parameters for each element (initially set to 0.03 Å^2) and the linear atomic scale factor (*delta1*) were allowed to be refined. If this resulted in an insufficient fit, possible secondary phases were added and refined in a similar way. In some cases, the Li isotropic displacement parameters refined to very large values (0.5 – 1 Å^2). If this

occurred, the Li isotropic displacement parameters were fixed to a lower value. Unphysical isotropic displacement parameters for Li have been reported previously and are an indication of disorder/partial occupancy or atomic substitution.[12] The refinements were conducted using the following structures for the fittings: $Ba_8Ga_{14.9}Sn_{31.1}$ ($I\bar{4}3m$, **Table S1-2**), β-Sn ($I4_1/amd$, ICSD-252800),[13], LiSn ($P2/m$, ICSD- 104782),[14] Li_7Sn_3 ($P2_1/m$, ICSD-104785),[15] and Li_7Sn_2 ($Cmmm$, ISCD-104784).[16]

The relative phase content determined from the PDFgui refinement is calculated by normalizing the percentage of each phase, with the phase written in whole atom compositions. For example, the refinement results for the data shown in **Figure S6a** were 70% of $Li_{0.5}Sn_{0.5}$ and 30% of $Li_{0.7}Sn_{0.3}$, which we designate as 70% of LiSn and 30% of Li_7Sn_3.

1.5. Powder X-ray Diffraction (XRD)

Powder X-ray diffraction was performed using a Bruker D8 diffractometer with Cu X-ray source (Kα1, Kα2) operated at 40 kV and 40 mA with standard Bragg-Brentano diffraction geometry. Powder X-ray diffraction (PXRD) for the Rietveld refinement was performed with a Malvern PANalytical Aeris research edition powder diffractometer with Cu X-rays (Kα1, Kα2) operated at 40 kV and 40 mA with standard Bragg-Brentano diffraction geometry and Pixel3D detector. Rietveld refinement was performed with Jana2006.[17] The peak shapes were described by the pseudo-Voigt function background fit with Legendre polynomials.

1.6. Scanning Electron Microscopy

Scanning electron microscopy (SEM) imaging was performed using an XL30 ESEM-FEG microscope and a 20 kV electron beam. The electrodes were cut from copper foil and mounted on SEM stubs with carbon tape. Energy dispersive X-ray spectroscopy (EDS) measurements were taken at 20 kV with a 2.4 nA spot size

1.7. Density Functional Theory (DFT) Calculations

The first-principles DFT calculations were performed to explore Li insertion and migration in the type VIII clathrate using a similar manner as in our previous work[18–20]. The calculations were carried out using the VASP code[21,22], the PBE functional[23], and projector augmented wave (PAW) potentials with a plane wave basis set[22]. In the PAW potentials, the Sn 5p and 4d, Ba 5s, 5p, and 6s, Ga 4p and 3d and Li 1s and 2s electrons were treated as valence electrons. The kinetic energy cutoff for the plane wave basis set was chosen to be 400 eV and the reciprocal space was sampled with the Monkhorst pack meshes 3 × 3 × 3 centered at Γ. The $Ba_8Ga_{15}Sn_{31}$ structure was created by starting with Ba_8Sn_{46}, using the atomic positions obtained from the single crystal refinement. Then, the Sn atoms were replaced with Ga using the fractional occupancies derived from the single crystal refinement. The distribution of Ga on the four Wyckoff sites (24*g* : 12*d* : 8*c* : 2*a*) was chosen to be 7 : 3 : 5 : 0 while minimizing Ga–Ga bonds. The convergence criteria for the electronic and ionic relaxations were set to be 0.01 and 0.1 meV, respectively. The geometric optimization was performed in two steps. First, the unit cell volume was optimized without any constraints on the lattice parameters. Then the relaxed lattice constant, taken from the relaxed volume, was used in a second step where only ionic relaxation was allowed with a fixed lattice constant determined from the first step. The crystal structures with the ionic positions of the second step are reported. The CIF of the DFT-calculated structure of $Ba_8Ga_{15}Sn_{31}$ is available as a supporting information file.

The formation energy was calculated as described previously.[19,24] The elemental energies of Ba, Sn, and Ga were –1.923 eV/atom, –3.972 eV/atom, and –3.0281 eV/atom, respectively. The elemental energies of bcc Ba and β-Sn were calculated with a 400 eV energy cutoff and a 12 x 12 x 12 Kpoint grid. The energy of elemental Ga was taken from the Materials Project (mp-142).[25] The formation energy was determined using equation 3:

$$E_{form} = \frac{E(Ba_x Ga_y Sn_z) - xE(Ba) - yE(Ga) - zE(Sn)}{x+y+z} \qquad (3)$$

where $E(Ba_xGa_ySn_z)$ is the calculated energy of the Ba-Ga-Sn clathrate, $E(Ba)$,$E(Ga)$ and $E(Sn)$ are the total energy per atom of bulk Ba, Ga, and Sn, respectively. The Gibbs free energy change of reaction (ΔG_r) and the average voltage were calculated as described previously.[18–20] The formulas used for calculating the Gibbs free energy change and average voltage for insertion of a single Li atom into $Ba_8Ga_{15}Sn_{31}$ are shown in equation (4) and (5), respectively:

$$\Delta G_r = E(LiBa_8Ga_{15}Sn_{31}) - E(Ba_8Ga_{15}Sn_{31}) - E(Li) \qquad (4)$$

$$V(x) = -\Delta G_r/e^- \qquad (5)$$

where $E(LiBa_8Ga_{15}Sn_{31})$ and $E(Ba_8Ga_{15}Sn_{31})$ are the total free energies for the lithiated and unlithiated clathrate systems and $E(Li)$ is the energy per atom in Li metal. ΔG_r is equal to $\Delta E_r + P\Delta V_r - T\Delta S_r$ and the P and T terms are predicted to be sufficiently small compared to internal energy of ΔE_r so that ΔGr is approximately the energy from the DFT calculation. The value for $E(Li)$ is –1.904 eV/atom which was calculated with the bcc Li structure a 400 eV energy cutoff with 12 x 12 x 12 Kpoint grid. A negative ΔG_r (*i.e.* positive voltage) represents a spontaneous reaction relative to Li metal, suggesting the feasibility of lithiation in a half cell with Li metal as the counter electrode. All crystal structure figures were created with Diamond 4.5.3.

The climbing image nudged elastic band (NEB) method was used to calculate the Li migration barriers[26]. For the NEB calculations Monkhorst pack meshes 3 × 3 × 3 centered at Γ were used. All NEB calculations used a linear interpolation as the starting configurations with 7 intermediate images between the beginning and ending images. The images were converged until the force on each image was below 0.03 eV/Å.

1.8. Synthesis and Characterization of Type I Clathrate $K_8Li_xGe_{46-x}$

The $K_8Li_xGe_{46-x}$ clathrate was synthesized using Liang's method.[27] The as-made sample was ground by hand and then washed three times with water. After heating overnight at 50 °C under vacuum to remove the moisture, the powder was prepared into slurries with 10 wt% carbon black and 10 wt%

PVDF in NMP solvent. The slurries were stirred overnight and coated onto Cu foil current collectors using a Meyer rod, and then heated at 120 ^{o}C to remove the solvent. Half-cells were assembled using Li metal as the counter electrode in the glovebox. Electrochemical testing was performed using a Biologic VMP3 galvanostat/potentiostat. Galvanostatic measurements were performed from 0.01 – 2.5 V vs. Li/Li$^+$ range using a current density of 25 mA/g, while potentiostatic measurements were performed within the same voltage range using a current density of 100 mA/g.

2. Supporting Tables

Table S1. Crystallographic data for $Ba_8Ga_{14.9}Sn_{31.1}$ from single crystal XRD (CIF has been deposited with the CCDC database with a reference number 2071610).

**Empirical Formula**	$\mathbf{Ba_8Ga_{14.9}Sn_{31.1(4)}}$
Formula Weight	5829.30
Temperature (K)	200(2)
Radiation, λ	MoK_a, 0.71073Å
Crystal System	Cubic
Space Group, Z	$I\bar{4}3m$ (no. 217), Z = 1
a/Å	11.5887(9)
V/Å^3	1556.3(4)
$\rho_{calc.}$/ g cm^{-3}	6.22
μ/cm^{-1}	234.9
R_1 ($I>2\sigma_I$)^a	0.0192
wR_2 ($I>2\sigma_I$)^a	0.0330
R_1 (all data)	0.0216
wR_2 (all data)	0.0335
Largest diff. Peak & hole / e$^-$ Å^{-3}	0.65 / -0.55
Goodness of Fit (Gof)	1.143

^{a} $R_1 = \sum||F_o| - |F_c||/\sum|F_o|$; $wR_2 = [\sum[w(F_o^2 - F_c^2)^2]/\sum[w(F_o^2)^2]]^{1/2}$, where $w = 1/[\sigma^2F_o^2 + (0.0084\cdot P)^2 + (2.831\cdot P)]$, and $P = (F_o^2 + 2F_c^2)/3$.

Table S2. Atomic coordinates, occupancies, and equivalent isotropic displacement parameters ($U_{eq}{}^a$) of $Ba_8Ga_{14.9}Sn_{31.1}$ from single crystal XRD refinement

**Atom**	**Wyckoff Site**	***x***	***y***	***z***	***Occupancy***	$\mathbf{U_{eq}}$ **(Å^2)**
Ba	8*c*	0.31513(7)	*x*	*x*	1	0.0345(5)
Sn1	24*g*	0.08429(5)	*x*	0.35137(6)	0.70(1)	0.0141(2)
Ga1	24*g*	0.08429(5)	*x*	0.35137(6)	0.30(1)	0.0141(2)
Sn2	12*d*	1/4	1/2	0	0.85(2)	0.0182(4)
Ga2	12*d*	1/4	1/2	0	0.15(2)	0.0182(4)
Sn3	8*c*	0.13423(7)	*x*	*x*	0.33(2)	0.0126(5)
Ga3	8*c*	0.13423(7)	*x*	*x*	0.67(2)	0.0126(5)
Sn4	2*a*	0	0	0	0.78(3)	0.0100(8)
Ga4	2*a*	0	0	0	0.22(3)	0.0100(8)

^{a} U_{eq} is defined as one third of the trace of the orthogonalized U_{ij} tensor.

Table S3. Refinement parameters for PDF refinement of pristine clathrate sample fit to type VIII $Ba_8Ga_{15}Sn_{31}$ and β-Sn. The refinement plot is presented in **Figure 2c**.

	**Pristine $Ba_8Ga_{15}Sn_{31}$**	
**Phase**	Type VIII $Ba_8Ga_{15}Sn_{31}$	β-$Sn_{0.93}Ga_{0.07}$
**Space Group**	$I\bar{4}3m$	$I4_1/amd$
**Phase Fraction**	0.97(3)	0.03(3)
**Lattice Parameters (Å)**	a = 11.60(1)	*a* = 5.7(1)<br>*c* = 3.2(1)
**Delta 1**	1.9	2.00*
**Atomic displacement parameters (U_{11}= U_{22}= U_{33}, Å^2)**	Ba = 0.04(2)<br>Ga/Sn = 0.020(6)	Sn = 0.014*
R_w	0.151	

*These parameters were fixed during the refinement due to resulting in unphysical values if not fixed.

Table S4. The voltage and capacity corresponding to the lithiated type VIII $Ba_8Ga_{15}Sn_{31}$ and β-Sn samples used for PDF measurements (corresponding to the points indicated in **Figure 3**). The number of Li inserted was normalized to the amount of Sn or (Ga + Sn) in the starting compound.

**Electrode**	**# of Li inserted**	**Voltage (V vs. Li/Li$^+$)**	**Capacity (mAh/g)**
**$Ba_8Ga_{15}Sn_{31}$**	1.5 Li	0.213	320
	2.0 Li	0.171	427
	3.2 Li	0.010	683
**β-Sn**	1.5 Li	0.440	338
	2.0 Li	0.387	451
	2.5 Li	0.236	564
	3.4 Li	0.010	767

Table S5. PDF refinement parameters of best fits for β-Sn, $Li_{1.5}Sn$, $Li_{2.0}Sn$, $Li_{2.5}Sn$, $Li_{3.4}Sn$ samples. Plots of the refinements are presented in **Figure S6**.

	**Pristine**	**$Li_{1.5}Sn$**		**$Li_{2.0}Sn$**		**$Li_{2.5}Sn$**	**$Li_{3.4}Sn$**	
**Phase**	β-Sn<br>$I4_1/amd$	LiSn<br>$P2/m$	Li_7Sn_3<br>$P2_1/m$	LiSn<br>$P2/m$	Li_7Sn_3<br>$P2_1/m$	Li_7Sn_3<br>$P2_1/m$	Li_7Sn_3<br>$P2_1/m$	Li_7Sn_2<br>$Cmmm$
**Phase Fraction**	1	0.7(1)	0.3(1)	0.18(5)	0.82(5)	1	0.53(8)	0.47(8)
**Lattice Parameters (Å)**	*a* = 5.833(5)<br>*c* = 3.180(6)	*a* = 5.19(3)<br>*b* = 3.18(2)<br>*c* = 7.72(7)<br>β=104.5°	*a* = 8.5(2)<br>*b* = 4.7(1)<br>*c* = 9.4(3)<br>β=105.95°	*a* = 5.18(7)<br>*b* = 3.19(4)<br>*c* = 7.7(2)<br>β=104.5°	*a* = 8.59(5)<br>*b* = 4.71(3)<br>*c* = 9.46(7)<br>β=105.95°	*a* = 8.60(3)<br>*b* = 4.70(2)<br>*c* = 9.43(4)<br>β=105.95°	*a* = 8.56(7)<br>*b* = 4.71(4)<br>*c* = 9.48(8)<br>β=105.95°	*a* = 9.7(2)<br>*b* = 13.9(2)<br>*c* = 4.72(8)
**Delta 1**	2.2	1.8	1.5	2.2	2.5	2.4	1.1	2.8
**Atomic displacement parameters** **(U_{11}= U_{22}= U_{33}, Å^2)**	Sn = 0.023(3)	Li = 0.023(6)<br>Sn = 0.016(5)	Li = 0.076*<br>Sn = 0.011(2)	Li = 0.04(23)<br>Sn = 0.013(1)	Li = 0.2(3)<br>Sn = 0.017(3)	Li = 0.2(2)<br>Sn =0.020(3)	Li = 0.1*<br>Sn = 0.018(6)	Li = 0.1*<br>Sn = 0.02(2)
**R_w**	0.102	0.159		0.115		0.139	0.199	

*Refinement of the ADP for Li in both cases resulted in unreasonably high values so it was fixed.

Table S6. PDF refinement parameters of fits for the lithiated clathrate samples to different phase combinations. Plots of the refinements are presented in **Figure S7**.

	**$Li_{1.5}Ba_{0.17}Ga_{0.33}Sn_{0.67}$**		**$Li_{2.0}Ba_{0.17}Ga_{0.33}Sn_{0.67}$**	**$Li_{3.2}Ba_{0.17}Ga_{0.33}Sn_{0.67}$**
**Phase**	Type VIII<br>$Ba_8Ga_{15}Sn_{31}$<br>$I\bar{4}3m$	Li_7Sn_3<br>$P2_1/m$	Li_7Sn_3<br>$P2_1/m$	Li_7Sn_3<br>$P2_1/m$
**Phase Fraction**	0.30(9)	0.69(9)	1	1
**Lattice Parameters (Å)**	*a* = 11.60(3)	*a* = 8.5(1)<br>*b* = 4.68(7)<br>*c* = 9.5(1)<br>β=105.95°	*a* = 8.4(1)<br>*b* = 4.70(6)<br>*c* = 9.5(1)<br>β=105.95°	*a* = 8.5(1)<br>*b* = 4.69(5)<br>*c* = 9.6(1)<br>β=105.95°
**Delta 1**	2.1	2.00*	2.00*	2.00*
**Atomic displacement parameters (U_{11}= U_{22}= U_{33}, Å^2)**	Ba = 0.04*<br>Ga/Sn = 0.0236*	Li = 0.05*<br>Sn = 0.025*	Li = 0.02*<br>Sn = 0.02*	Li = 0.02*<br>Sn = 0.02*
**R_w**	0.493		0.820	0.855

*These parameters were fixed during the refinement due to resulting in unphysical values if not fixed.

Table S7. Lattice parameters of the DFT calculated structures ($LiBa_8Ga_{15}Sn_{31}$) shown in **Figure 8**.

**Li Position**	**Lattice Parameter (Å)**	**Difference (Å)**
Unlithiated Clathrate	11.838	n/a
1 (Void Center)	11.893	0.055
2 (Six-membered Ring)	11.880	0.042
3 (Six-membered Ring)	11.878	0.04
4 (Six-membered Ring)	11.874	0.036

3. Supporting Figures

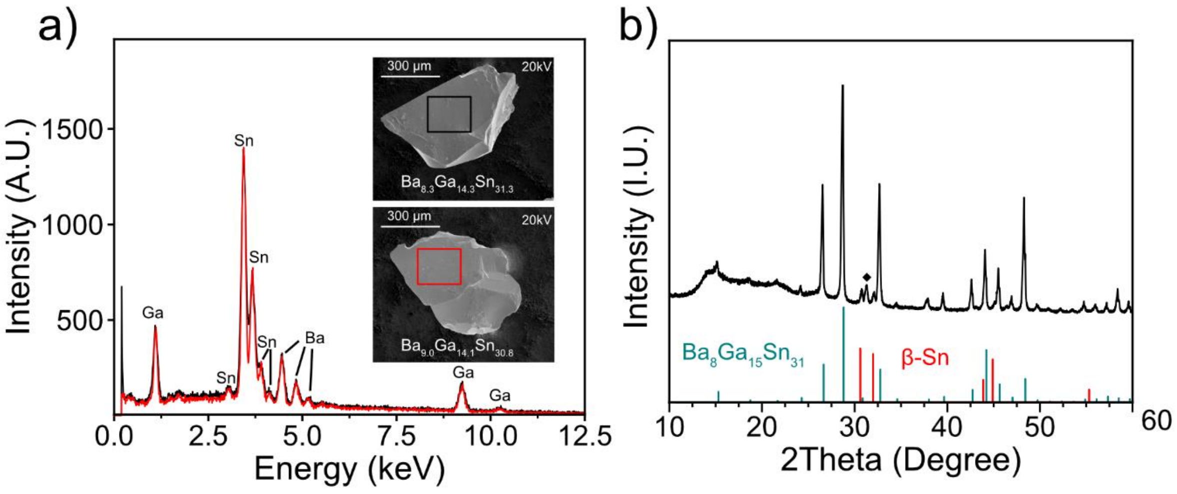

Figure S1. **(a)** SEM images and EDS spectra (20 kV) of two single crystal particles from the as-synthesized type VIII $Ba_8Ga_{15}Sn_{31}$ clathrate sample, where the black and red boxes represent the areas where the EDS spectra were taken. The compositions denoted in each SEM image were calculated from the EDS collection software. **(b)** Laboratory powder XRD pattern of crushed single crystals from the as-synthesized clathrate sample with the simulated diffraction patterns for type VIII $Ba_8Ga_{15}Sn_{31}$ (from single crystal diffraction, **Table S1-S2**) and β-Sn. The background from $15° < 2\theta < 25°$ is from the Kapton film that was used to protect the sample from oxidation during the measurement. The reflection marked with a diamond is not seen in the powder pattern of the electrode (**Figure 2b**) suggesting that this unknown phase might have reacted or dissolved during electrode preparation.

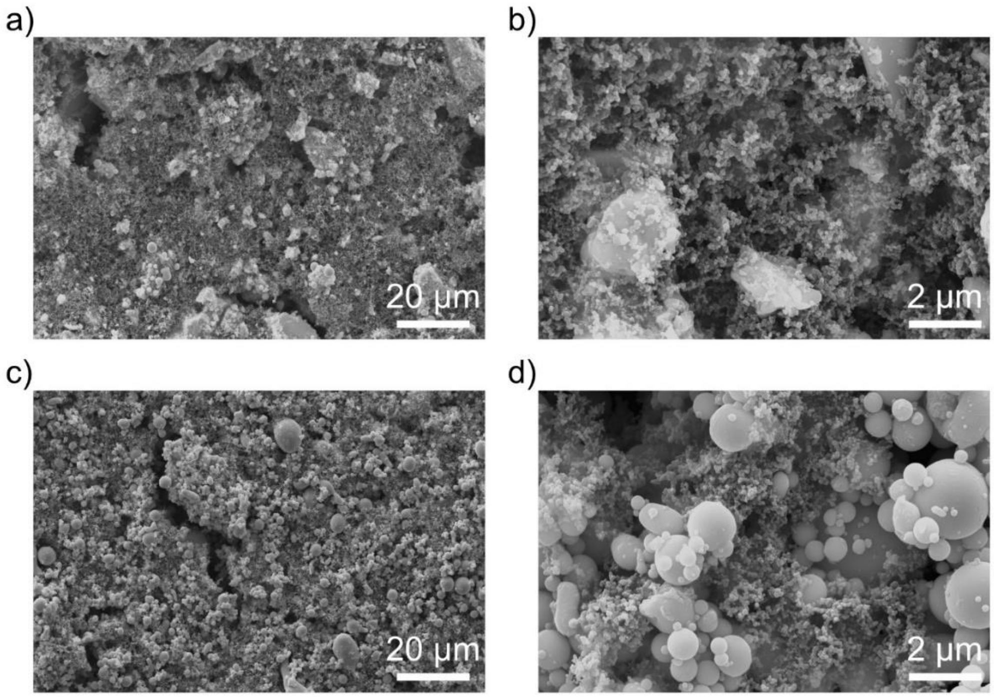

Figure S2. SEM images (secondary electron) of the composite electrodes made from **(a,b)** type VIII $Ba_8Ga_{15}Sn_{31}$ clathrate, and **(c,d)** β-Sn prior to electrochemical lithiation. The small, nanosized particles are the carbon black conducting additive used to improve electronic conductivity throughout the composite electrode.

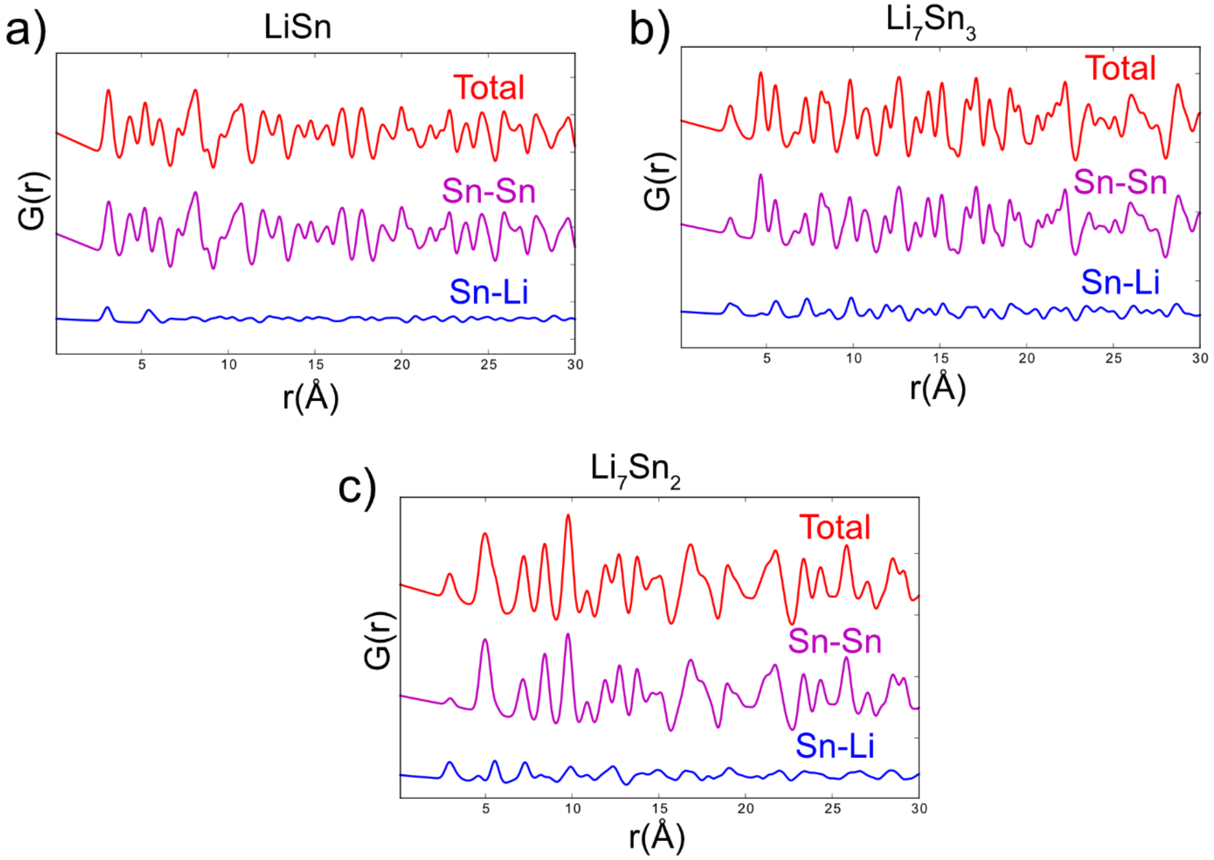

Figure S3. Calculated total and partial PDF patterns for **(a)** LiSn, **(b)** Li_7Sn_3, and **(c)** Li_7Sn_2.

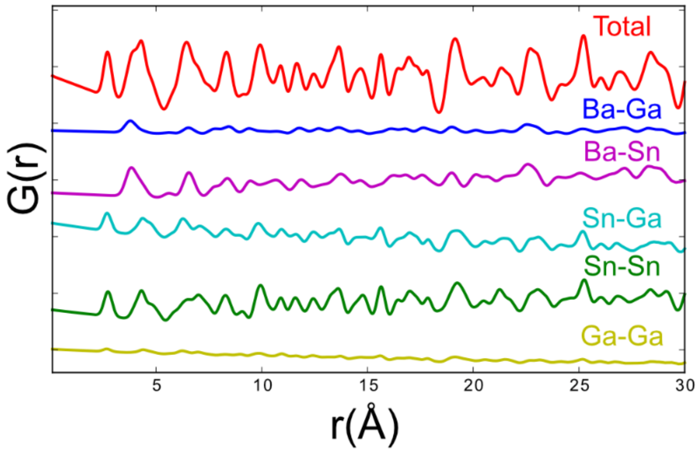

Figure S4. Calculated total and partial PDF patterns for type VIII clathrate $Ba_8Ga_{15}Sn_{31}$.

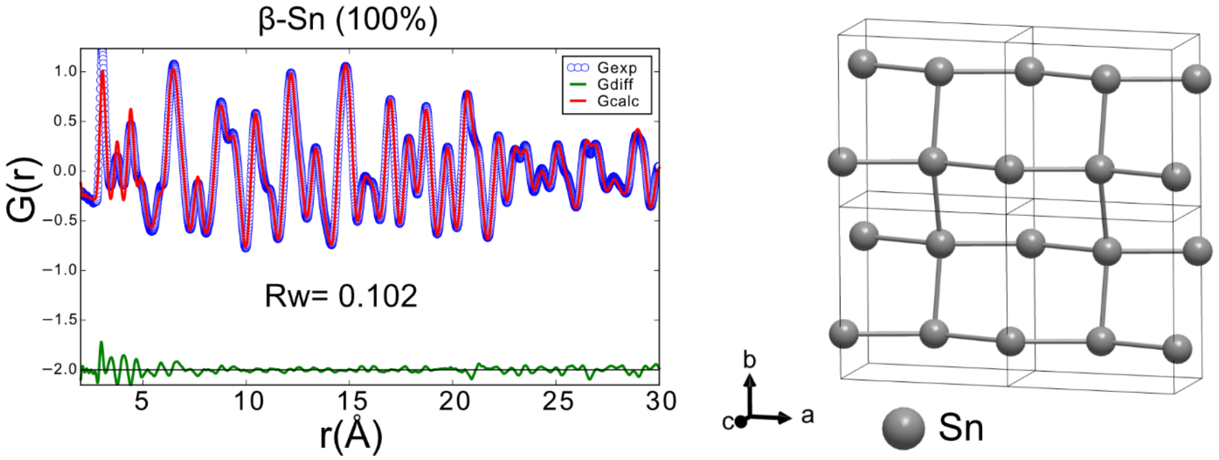

Figure S5. PDF refinement and crystal structure of pristine β-Sn

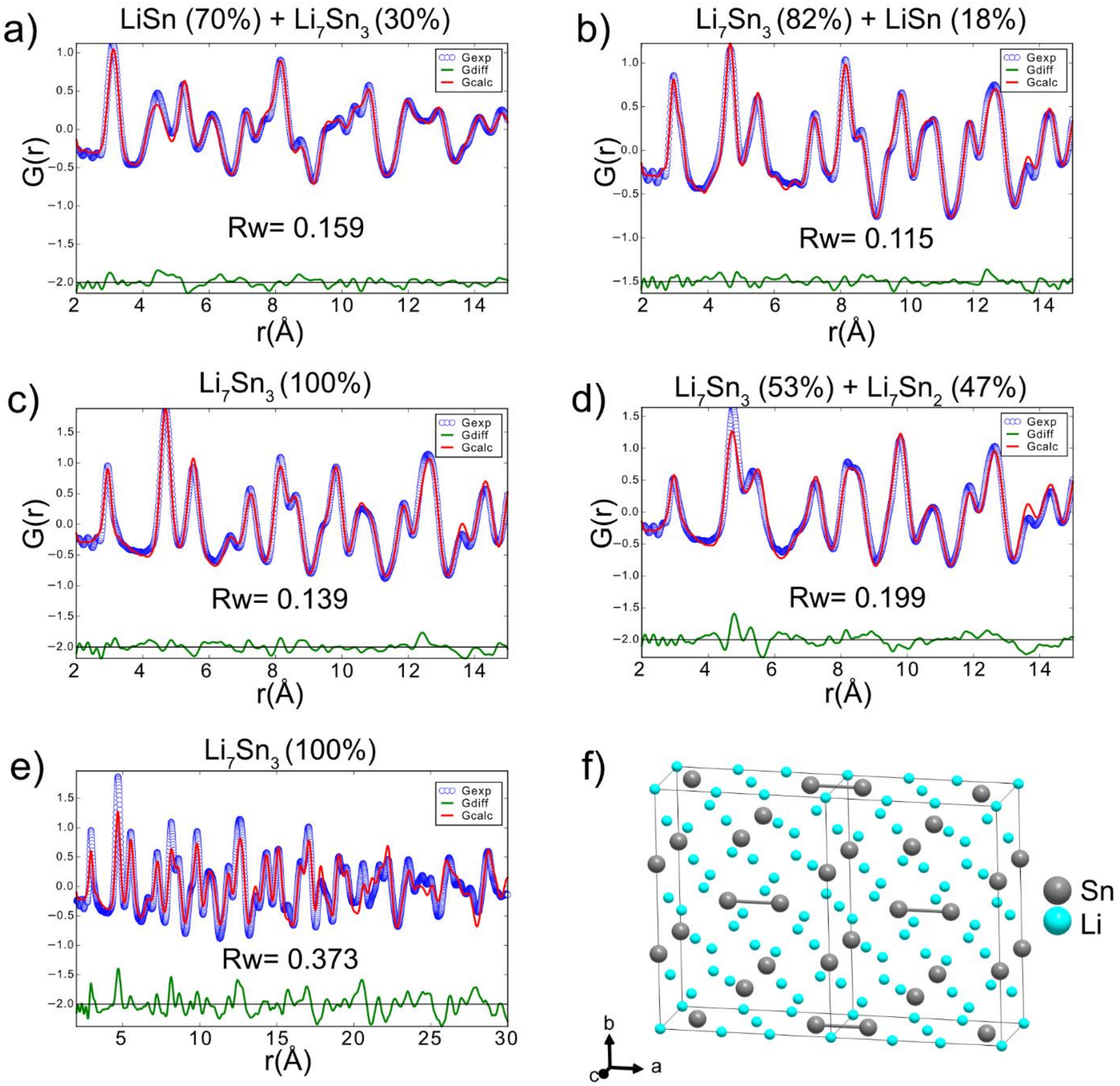

Figure S6. PDF refinements of the lithiated β-Sn PDF patterns at compositions of **(a)** $Li_{1.5}Sn$ **(b)** $Li_{2.0}Sn$, **(c)** $Li_{2.5}Sn$ and **(d)** $Li_{3.4}Sn$. **(e)** $Li_{2.5}Sn$ fit from 2 – 30 Å. The phases used in the fitting and corresponding mol% from the refinements are shown above each plot. **(f)** Crystal structure of Li_7Sn_2.

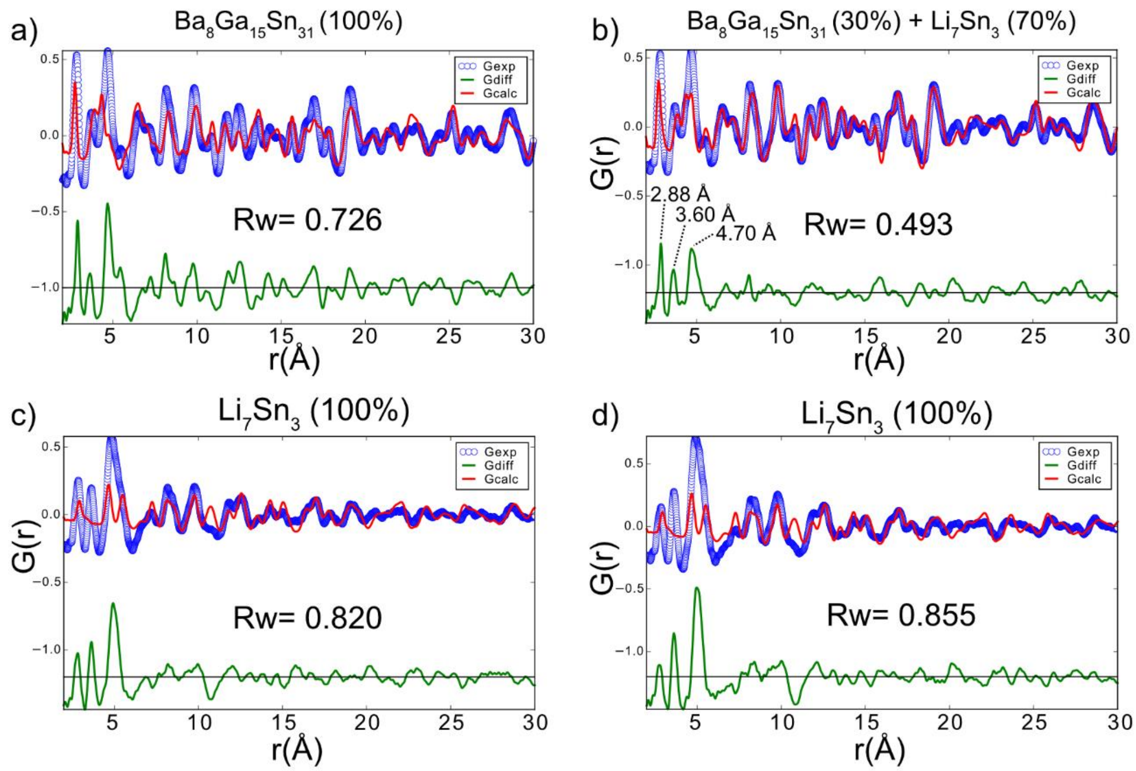

Figure S7. PDF refinements of the lithiated type VIII clathrate PDF patterns at composition of **(a),(b)** $Li_{1.5}Ba_{0.17}Ga_{0.33}Sn_{0.67}$, **(c)** $Li_{2.0}Ba_{0.17}Ga_{0.33}Sn_{0.67}$, and **(d)** $Li_{3.2}Ba_{0.17}Ga_{0.33}Sn_{0.67}$. The phases used in the fitting and corresponding mol% from the refinements are shown above each plot.

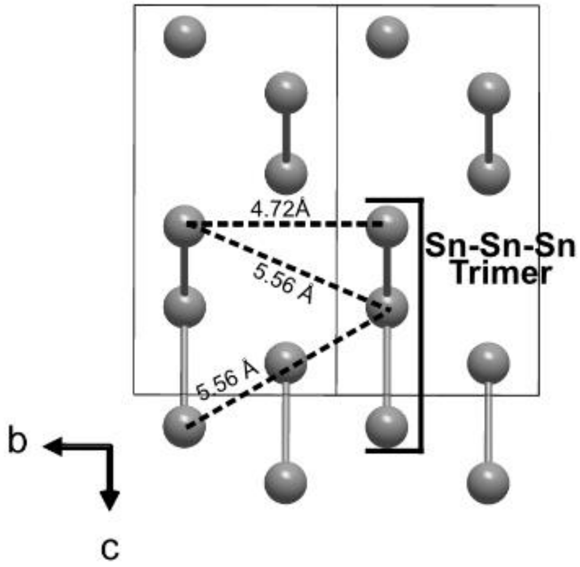

Figure S8. Crystal model schematic of Li_7Sn_3 showing only the Sn atoms viewed down the [100] direction. The dashed lines show the distances that correspond to the two split peaks in the PDF of Li_7Sn_3 associated with two different next-nearest neighbor Sn-Sn correlations.

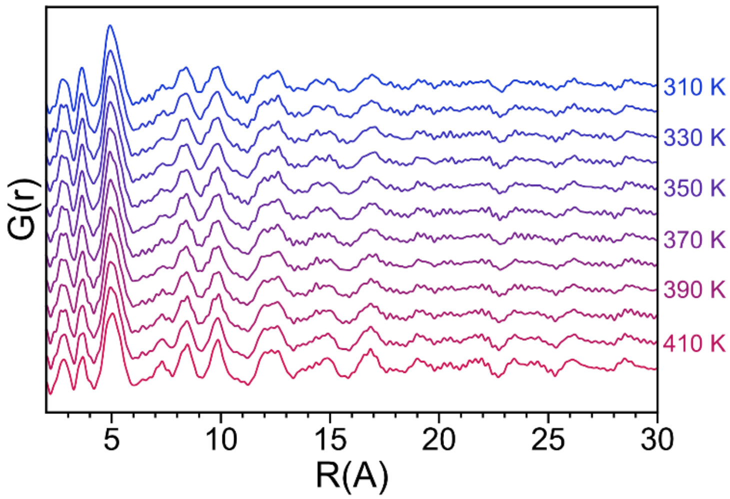

Figure S9. Variable temperature (310 K- 420 K) PDF of the fully lithiated type VIII clathrate ($Li_{3.2}Ba_{0.17}Ga_{0.33}Sn_{0.67}$). An interval of 10 K and a 5 minute hold at each temperature was used.

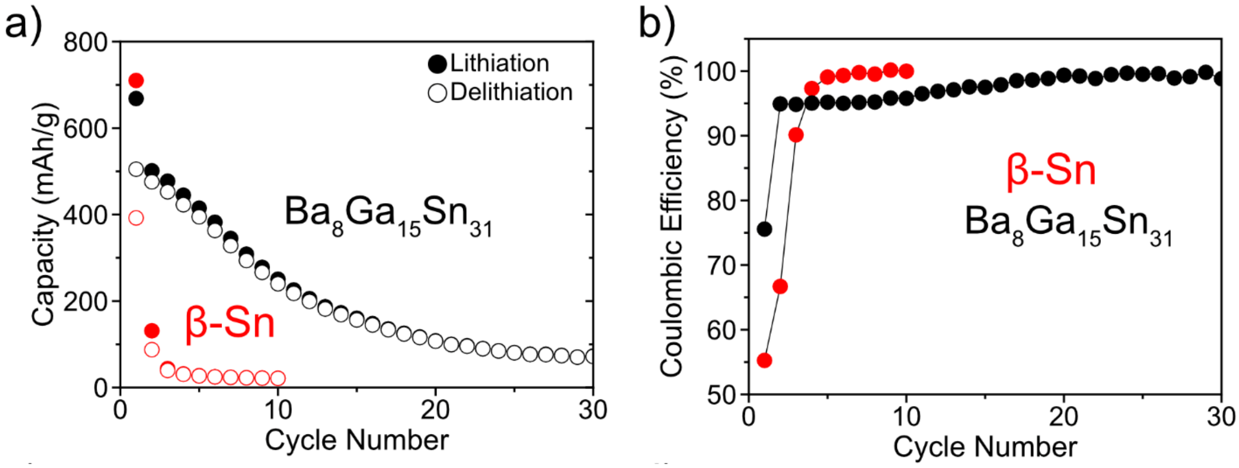

Figure S10. **(a)** Capacity and **(b)** Coulombic efficiency vs. cycle number for β-Sn and type VIII $Ba_8Ga_{15}Sn_{31}$ clathrate electrodes in half-cells cycled at 12.5 mAh/g with a voltage range of 0.01 – 2.5 V vs. Li/Li^+.

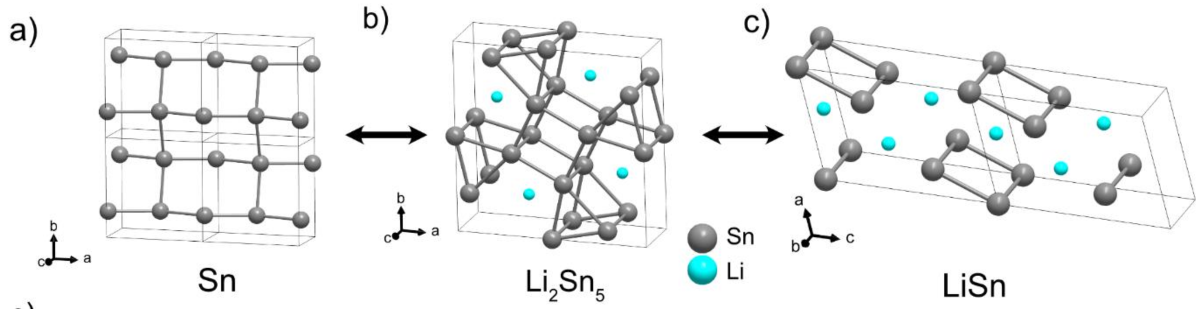

Figure S11. Crystal structures of **(a)** β-Sn, **(b)** Li_2Sn_5 and **(c)** LiSn. The common square Sn unit between the three structures has been suggested to make these phase transformations kinetically facile.

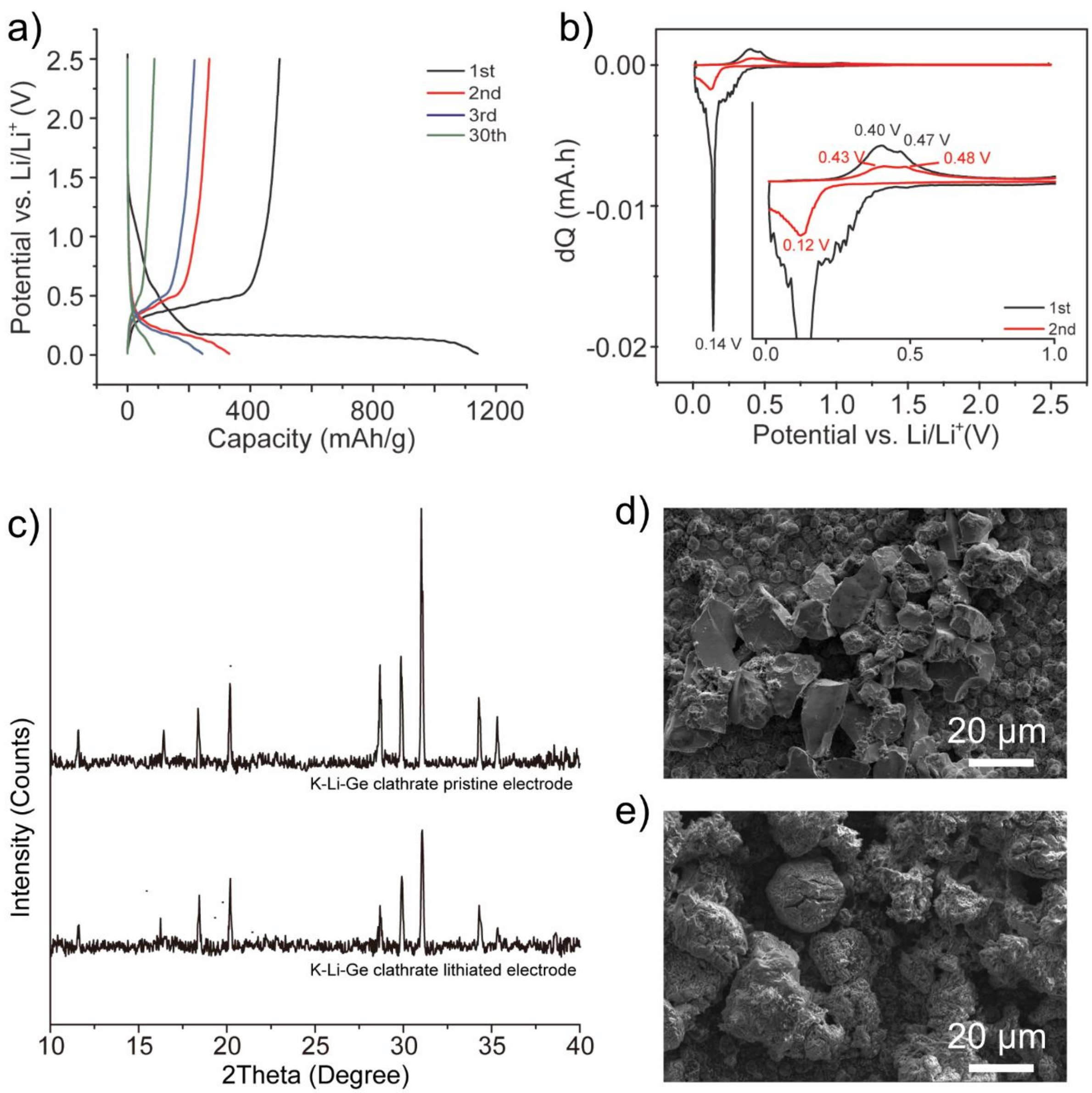

Figure S12. (a) Galvanostatic cycling of $K_8Li_xGe_{46-x}$ electrodes at 25 mA/g. **(b)** dQ plot derived from potentiostatic measurements with a current limit of a 100 mA/g. **(c)** PXRD of the clathrate electrodes before and after full lithiation. SEM images of the clathrate electrode **(d)** before and **(e)** after lithiation.

4. References

(1) Schäfer, M. C.; Yamasaki, Y.; Fritsch, V.; Bobev, S. Ternary Compounds in the Sn-Rich Section of the Ba–Ga–Sn System: $Ba_8Ga_{16-x}Sn_{30+x}$ ($1.1 \leq x \leq 2.8$) Clathrates of Type-I and Type-VIII, and $BaGa_{2-x}Sn_{4+x}$ ($x \approx 0.2$) with a Clathrate-like Structure. *Crystals* **2011**, *1*, 145–162.

(2) Bruker AXS Inc. *SAINT*; Bruker AXS Inc: Madison, Wisconsin, USA, 2014.

(3) Bruker AXS Inc. *SADABS*; Bruker AXS Inc: Madison, Wisconsin, USA, 2014.

(4) Sheldrick, G. M. SHELXT - Integrated Space-Group and Crystal-Structure Determination. *Acta Crystallogr. Sect. A Found. Crystallogr.* **2015**, *71*, 3–8.

(5) Dolomanov, O. V.; Bourhis, L. J.; Gildea, R. J.; Howard, J. A. K.; Puschmann, H. OLEX2: A Complete Structure Solution, Refinement and Analysis Program. *J. Appl. Crystallogr.* **2009**, *42*, 339–341.

(6) Sheldrick, G. M. Crystal Structure Refinement with SHELXL. *Acta Crystallogr. Sect. C Struct. Chem.* **2015**, *71*, 3–8.

(7) Gelato, L. M.; Parthé, E. STRUCTURE TIDY– a Computer Program to Standardize Crystal Structure Data. *J. Appl. Crystallogr.* **1987**, *20*, 139–143.

(8) Juhás, P.; Davis, T.; Farrow, C. L.; Billinge, S. J. L. PDFgetX3: A Rapid and Highly Automatable Program for Processing Powder Diffraction Data into Total Scattering Pair Distribution Functions. *J. Appl. Crystallogr.* **2013**, *46*, 560–566.

(9) Yang, X.; Juhas, P.; Farrow, C. L.; Billinge, S. J. L. XPDFsuite: An End-to-End Software Solution for High Throughput Pair Distribution Function Transformation, Visualization and Analysis. **2014**, arXiv 1402.3163.

(10) Farrow, C. L.; Juhas, P.; Liu, J. W.; Bryndin, D.; Boin, E. S.; Bloch, J.; Proffen, T.; Billinge, S. J. L. PDFfit2 and PDFgui: Computer Programs for Studying Nanostructure in Crystals. *J. Phys. Condens. Matter* **2007**, *19*.

(11) Borkiewicz, O. J.; Shyam, B.; Wiaderek, K. M.; Kurtz, C.; Chupas, P. J.; Chapman, K. W. The AMPIX Electrochemical Cell: A Versatile Apparatus for in Situ X-Ray Scattering and Spectroscopic Measurements. *J. Appl. Crystallogr.* **2012**, *45*, 1261–1269.

(12) Osman, H. H.; Bobev, S. Synthesis, Structural Characterization and Chemical Bonding of $Sr_7Li_6Sn_{12}$ and Its Quaternary Derivatives with Eu and Alkaline Earth Metal (Mg, Ca, Ba) Substitutions. A Tale of Seven Li-Containing Stannides and Two Complex Crystal Structures. *Eur. J. Inorg. Chem.* **2020**, *2020*, 1979–1988.

(13) Allison, M. C.; Avdeev, M.; Schmid, S.; Liu, S.; Söhnel, T.; Ling, C. D. Synthesis, Structure and Geometrically Frustrated Magnetism of the Layered Oxide-Stannide Compounds $Fe(Fe_{3-x}Mn_x)Si_2Sn_7O_{16}$. *Dalt. Trans.* **2016**, *45*, 9689–9694.

(14) Müller, W.; Schäfer, H. Die Kristallstruktur Der Phase LiSn:The Crystal Structure of

LiSn. *Zeitschrift fur Naturforsch. - Sect. B J. Chem. Sci.* **1973**, *28*, 246–248.

(15) Müller, W. Preparation Und Crystal Structure of Li_7Sn_3. *Zeitschrift für Naturforsch. B* **1974**, *29*, 304–311.

(16) Frank, U.; Müller, W.; Schäfer, H. Die Kristallstruktur Der Phase Li_7Sn_2. *Zeitschrift fur Naturforsch. - Sect. B J. Chem. Sci.* **1975**, *30*, 6–9.

(17) Petrícek, V.; Dušek, M.; Palatinus, L. Crystallographic Computing System JANA2006: General Features. *Zeitschrift fur Kristallographie*. 2014, pp 345–352.

(18) Peng, X.; Wei, Q.; Li, Y.; Chan, C. K. First-Principles Study of Lithiation of Type I Ba-Doped Silicon Clathrates. *J. Phys. Chem. C* **2015**, *119*, 28247–28257.

(19) Dopilka, A.; Zhao, R.; Weller, J. M.; Bobev, S.; Peng, X.; Chan, C. K. Experimental and Computational Study of the Lithiation of $Ba_8Al_yGe_{46-y}$ Based Type I Germanium Clathrates. *ACS Appl. Mater. Interfaces* **2018**, *10*, 37981–37993.

(20) Dopilka, A.; Peng, X.; Chan, C. K. Ab Initio Investigation of Li and Na Migration in Guest-Free, Type I Clathrates. *J. Phys. Chem. C* **2019**, *123*, 22812–22822.

(21) Kresse, G.; Furthmüller, J. Efficient Iterative Schemes for Ab Initio Total-Energy Calculations Using a Plane-Wave Basis Set. *Phys. Rev. B - Condens. Matter Mater. Phys.* **1996**, *54*, 11169–11186.

(22) Kresse, G.; Joubert, D. From Ultrasoft Pseudopotentials to the Projector Augmented-Wave Method. *Phys. Rev. B* **1999**, *59*, 1758–1775.

(23) Perdew, J. P.; Burke, K.; Ernzerhof, M. Generalized Gradient Approximation Made Simple. *Phys. Rev. Lett.* **1996**, *77*, 3865–3868.

(24) Wagner, N. A.; Raghavan, R.; Zhao, R.; Wei, Q.; Peng, X.; Chan, C. K. Electrochemical Cycling of Sodium-Filled Silicon Clathrate. *ChemElectroChem* **2014**, *1*, 347–353.

(25) Jain, A.; Ong, S. P.; Hautier, G.; Chen, W.; Richards, W. D.; Dacek, S.; Cholia, S.; Gunter, D.; Skinner, D.; Ceder, G.; Persson, K. A. Commentary: The Materials Project: A Materials Genome Approach to Accelerating Materials Innovation. *APL Mater.* **2013**, *1*.

(26) Henkelman, G.; Uberuaga, B. P.; Jónsson, H. Climbing Image Nudged Elastic Band Method for Finding Saddle Points and Minimum Energy Paths. *J. Chem. Phys.* **2000**, *113*, 9901–9904.

(27) Liang, Y.; Schnelle, W.; Veremchuk, I.; Böhme, B.; Baitinger, M.; Grin, Y. Synthesis and Thermoelectric Properties of the Clathrate-I Phase $K_8Li_xGe_{44-x/4}\square_{2-3x/4}$. *J. Electron. Mater.* **2015**, *44*, 4444–4451.
